# *U2af1*^S34F^ and *U2af1*^Q157R^ myeloid neoplasm-associated hotspot mutations induce distinct hematopoietic phenotypes in mice

**DOI:** 10.1038/s41375-026-02974-7

**Published:** 2026-05-06

**Authors:** Michael O. Alberti, Sridhar Nonavinkere Srivatsan, Jin Shao, Dennis L. Fei, Mengou Zhu, Claudia Cabrera Pastrana, Monique Chavez, Stefan P. Tarnawsky, Sarah Grieb, Timothy A. Graubert, Omar Abdel-Wahab, Matthew J. Walter

**Affiliations:** 1 Department of Pathology and Immunology, Washington University, St. Louis, MO USA; 2 Department of Pathology, University of Colorado Anschutz Medical Campus, Aurora, CO USA; 3 Department of Medicine, Washington University, St. Louis, MO USA; 4 Department of Medicine, Meyer Cancer Center, Weill Cornell Medicine, New York, NY USA; 5 Cancer Biology Section, Cancer Genetics Branch, National Human Genome Research Institute, Bethesda, MD USA; 6 Massachusetts General Hospital Cancer Center, Harvard Medical School, Boston, MA USA; 7 Memorial Sloan Kettering Cancer Center, New York, NY USA

**Keywords:** Cancer genetics, Cancer models, Myelodysplastic syndrome, Acute myeloid leukaemia, Myeloproliferative disease

## Abstract

Recurrent somatic mutations in the spliceosome genes *SF3B1*, *SRSF2*, and *U2AF1* are frequently identified in patients with myeloid neoplasms, such as myelodysplastic syndromes. We characterized the in vivo consequences of expressing two hotspot mutations in *U2AF1* that code for the S34F and Q157R substitutions. Our results indicate that the two mutations induce distinct hematopoietic phenotypes in mice, suggesting that the *U2AF1*^S34F^ and *U2AF1*^Q157R^ mutations should not be conflated as they may impact disease pathogenesis differently in patients. Mice expressing *U2af1*^S34F^ have a more severe reduction in their blood and bone marrow cell counts and reduced stem cell repopulating ability, compared to mice expressing *U2af1*^Q157R^. The expression and splicing of target genes are largely unique between the mutations, in both mouse and human samples, potentially driving the phenotypic differences induced by either mutation. The two mutations co-occur with different gene mutations in patients and are not equally represented across myeloid neoplasms, suggesting that multiple mechanisms likely drive U2AF1-mutant disease pathogenesis. Collectively, our results support that *U2AF1*^S34F^ and *U2AF1*^Q157R^ mutations induce distinct hematopoietic, gene expression, and RNA splicing phenotypes in vivo. Larger population studies will be needed to determine if these phenotypic changes translate into clinico-pathologic differences in patients, warranting separate classification.

## Introduction

Recurrent somatic mutations in a subset of spliceosome genes (*SF3B1*, *SRSF2*, and *U2AF1*) are frequently identified (30–60% depending on disease phenotype) in patients afflicted with myelodysplastic neoplasms (MDS), myeloproliferative neoplasms (MPN) such as myelofibrosis (MF), MDS/MPN overlap disorders such as chronic myelomonocytic leukemia (CMML), and secondary acute myeloid leukemia (sAML) [1–10]. These heterozygous and mutually exclusive mutations are enriched in hotspot codons in these 3’ splicing factor proteins resulting in aberrant alternative mRNA splicing in hematopoietic cells. However, each mutant protein predominantly affects a distinct set of alternatively spliced downstream target genes, suggesting that common downstream pathway alterations or cellular response to mutation expression, rather than specific shared splicing targets, may be responsible for MDS phenotypes, including dysplasia, ineffective hematopoiesis, and cytopenias [10–12].

*U2AF1* provides a unique opportunity to address this question because it has two hotspot positions (serine 34 [S34] and glutamine 157 [Q157]) that are each commonly mutated in MDS and are associated with unique mRNA splicing consequences [13, 14]. In addition, *U2AF1* S34 and Q157 codon mutations co-occur with mutations in different genes (e.g., *BCOR* and *ASXL1*, respectively) and patients with these mutations may have different hematopoietic phenotypes [15–18] highlighting that these mutations may induce distinct phenotypes. We asked if the splicing differences resulting from S34F and Q157R mutations were thus associated with different or similar effects on hematopoiesis. To do so, we characterized and compared an established conditional S34F knock-in mouse model [19] to a new Cre/*lox* conditional mouse model with the Q157R mutation knocked in to the endogenous *U2af1* locus, in order to directly study the hematopoietic phenotype, transcriptional, and mRNA splicing consequences of individual *U2AF1* gene mutations in vivo.

## Materials and methods

### Animal models and experimental details

*U2af1*^Q157R/+^ (MiniGene Q157R or “MGQ157R”) conditional knock-in mice were generated by Biocytogen (Waltham, MA). A full description of the targeting construct is provided in the Supplementary Methods. *U2af1*^S34F/+^ (“MGS34F”) conditional knock-in (Jackson Laboratory [JAX] Stock #032638, Bar Harbor, ME) [19] *U2af1*^fl/+^ conditional knockout (JAX Stock #037015), [20] *U2AF1*^WT^; *rtTA*, [21] *U2AF1*^S34F^; *rtTA* (JAX Stock #029784), [21] and *Mx1*-*Cre* (JAX Stock #003556) [22] mice are described elsewhere. B6.SJL-*Ptprc*^*a*^*Pepc*^*b*^/BoyCrCrl or “CD45.1” recipient mice were purchased from Charles River Laboratories (Stock #564). Heterozygous CD45.1/CD45.2 mice were bred by crossing C57BL/6J (B6; JAX Stock #000664) to B6.SJL-*Ptprc*^*a*^*Pepc*^*b*^/BoyJ (JAX Stock #002014). All mouse lines were on a B6 background. Genotyping primers are listed in Supplementary Table 1. The number of mice was based on the effect size and prior data in the literature. Analysis of mice was done between genotypes without randomization or blinding.

Details pertaining to bone marrow (BM) transplant, peripheral blood (PB) sampling and analysis, and flow cytometry setup and population gating are described in the Supplementary Methods.

### mRNA-sequencing (RNA-seq) and bioinformatics

BM myeloid progenitor (Kit^+^Lineage^−^Sca-1^−^; KL) cells were sorted into FACS buffer, and gDNA-depleted total RNA was purified from cell pellets using the NucleoSpin RNA Plus XS Micro Kit (Macherey-Nagel, Allentown, PA) in RNase-free water. RNA concentration and RIN were measured by Bioanalyzer (Agilent, Santa Clara, CA), and then cDNA libraries for RNA-seq were prepared by KAPA RNA HyperPrep Kit with RiboErase (Cat #KK8560/61; Roche, Indianapolis, IN). Detailed library preparation and bioinformatic analyses are described in Supplementary Methods.

Details pertaining to the bioinformatics and reanalysis of published MDS and AML RNA-seq datasets, analysis of *U2AF1* hotspot mutation co-occurrence in myeloid malignancies, and confirmation of splicing changes in mouse KL cell and MDS/sAML patient samples are also described in the Supplementary Methods. Clinical characteristics of patients who donated research samples are listed in Supplementary Table 2.

### Ethics approval and consent to participate

All methods were performed in accordance with the relevant guidelines and regulations. All patients provided written informed consent on a protocol approved by the Washington University in St. Louis (WUSTL) Human Studies Committee (Protocol # 201101915). Mouse experiments were performed per institutional guidelines for care and use of laboratory animals and approved by the WUSTL Institutional Animal Care and Use Committee (Protocol # 23-0434).

### Statistics

Data were analyzed and visualized using GraphPad Prism 10 software (Boston, MA). Statistical tests are described in each figure legend. *P* < 0.05 was considered statistically significant.

## Results

### Establishing a mouse model with conditional knock-in of the Q157R mutation at the *U2af1* locus

**Fig. 1:**
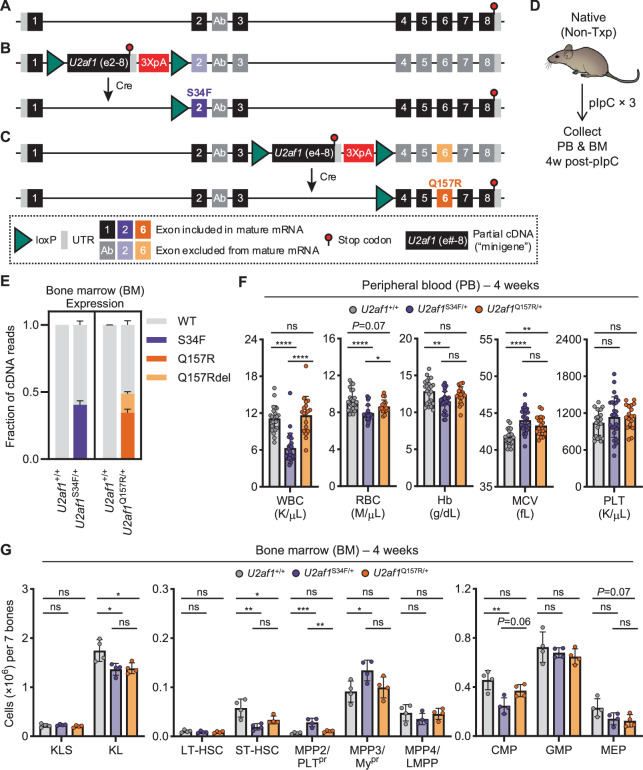
Characterization of native hematopoiesis in *U2af1*^S34F/+^ and *U2af1*^Q157R/+^ conditional knock-in mice. Diagrams of the wild-type (WT) mouse endogenous *U2af1* locus (**A**) and endogenous *U2af1* locus with conditional knock-in of either the S34F mutation in exon 2 (TCT > TTT) and upstream loxP flanked (floxed) MiniGene (MG; encoding WT *U2af1* exons 2-8) in intron 1 (“MGS34F”) (**B**) or Q157R mutation in exon 6 (CAG > CGG) and upstream floxed MG (encoding WT *U2af1* exons 4-8) in intron 3 (“MGQ157R”) (**C**). Cre-mediated recombination of the floxed MGS34F or MGQ157R alleles results in the removal of the WT MG cassette and conditional expression of *U2af1*^S34F^ or *U2af1*^Q157R^, respectively, from the mouse endogenous locus. 3XpA, three repeats of the SV40 late polyadenylation signal. See Supplementary Fig. 1A–C for targeting vectors and additional locus detail. **D** Non-transplant (native hematopoiesis) assay design. *U2af1*^+/+^, *U2af1*^S34F/+^, or *U2af1*^Q157R/+^ mice (all *Mx1*-*Cre*^+^) were treated with three doses of pIpC at 6–12 weeks of age. **E** Assessment of S34F and Q157R mRNA expression levels in BM KL cells at 4 weeks post-pIpC treatment. cDNA was prepared from KL cells for targeted NGS amplicon sequencing of the S34 (left) and Q157 (right) codons. The fraction of reads matching either WT or mutated alleles is plotted. *U2af1*^+/+^ mice were assessed for both S34F and Q157R/Q157Rdel alleles. The Q157R mutation in *U2af1* creates an alternative 5’ splice site that leads to expression of a minor *U2af1* isoform (termed “Q157Rdel”) with in-frame deletion of four amino acids immediately following the Q157R mutant codon. See also Supplementary Fig. 1D. *N* = 3 mice per genotype. **F** Complete blood count analysis (white blood cell [WBC], red blood cell [RBC], and platelet [PLT] counts, Hb [haemoglobin], and RBC mean corpuscular volume [MCV]) of PB samples from mice at 4 weeks post-pIpC. *N* = 18–26 mice per genotype, pooled from five independent experiments. **G** Absolute cell counts of BM hematopoietic stem and progenitor cell (HSPC) populations (KLS [Kit^+^Lineage^−^Sca-1^+^], KL [Kit^+^Lineage^−^Sca-1^−^], long- and short-term HSC [LT-HSC and ST-HSC], multipotent progenitors [MPP2, MPP3, and MPP4], common myeloid progenitors [CMP], granulocyte-macrophage progenitors [GMP], and megakaryocyte-erythrocyte progenitors [MEP]) were determined by flow cytometric analysis at 4 weeks post-pIpC. *N* = 4 mice per genotype. See also Supplementary Fig. 1E–G. Results represent the mean ± standard deviation (SD) (**E**–**G**). One-way analysis of variance (ANOVA) with Tukey multiple comparison correction (**F**, **G**) was used for the comparison of groups. *P < 0.05; **P < 0.01; ***P < 0.001; ****P < 0.0001. ns, not significant (or labeled if *P* < 0.10).

A conditional (Cre/*lox*-mediated) knock-in of the S34F mutation at the *U2af1* locus (MGS34F or *U2af1*^S34F/+^) was previously generated (Fig. 1A, B and Supplementary Fig. 1A) [19]. To allow for direct comparison with the MGS34F mouse, a similar strategy was used to generate a conditional (Cre/*lox*-mediated) Q157R mutant allele at the endogenous *U2af1* locus (MGQ157R or *U2af1*^Q157R/+^) of B6 mice (Fig. 1C and Supplementary Fig. 1B). Successful introduction of the targeting vector at the *U2af1* locus was confirmed by Southern blot (Supplementary Fig. 1C). To confirm Cre/*lox*-mediated hematopoietic expression of *U2af1*^Q157R^ mRNA and assess the short-term effects of U2AF1^Q157R^ in a non-transplant model (i.e., native hematopoiesis), we crossed heterozygous *U2af1*^Q157R/+^ mice to *Mx1-Cre* transgenic mice (Fig. 1D–G and Supplementary Fig. 1D–I). *Mx1-Cre* is expressed in hematopoietic lineage cells following administration of polyinosinic-polycytidylic acid (pIpC) [22].

Four weeks after pIpC treatment of *U2af1*^Q157R/+^; *Mx1-Cre* mice, the *U2af1* wild-type (WT) and Q157R alleles were expressed at similar levels in BM myeloid progenitor (KL) cells by targeted NGS amplicon sequencing of cDNA (Fig. 1E). As expected, the WT and S34F alleles were also expressed at similar levels in BM KL cells from *U2af1*^S34F/+^; *Mx1-Cre* mice and only the WT allele was detected in *U2af1*^+/+^; *Mx1-Cre* control mice (Fig. 1E). It was previously reported that the Q157R mutation in *U2AF1* creates an alternative 5’ splice site that leads to expression of a minor *U2AF1* isoform (termed “Q157Rdel”) with in-frame deletion of four amino acids immediately following the Q157R mutant codon [23]. The *U2af1*^Q157R/+^ mouse model recapitulates expression of the Q157Rdel isoform in BM KL cells (Fig. 1E and Supplementary Fig. 1D).

### U2AF1^S34F^ and U2AF1^Q157R^ cause different hematopoietic changes in mice

To determine if S34F and Q157R result in similar short-term effects on native hematopoiesis, we performed complete blood counts and flow cytometric analysis on PB samples from *U2af1*^Q157R/+^, *U2af1*^S34F/+^, and *U2af1*^+/+^ control mice (all *Mx1-Cre*^+^) four weeks after pIpC treatment. Consistent with previous characterization, [19] *U2af1*^S34F/+^ mice had no change in platelet counts, modestly reduced red blood cell (RBC) counts and hemoglobin levels (with elevated mean corpuscular volume [MCV]), and markedly reduced white blood cell (WBC) counts compared to *U2af1*^+/+^ mice (Fig. 1F). Flow cytometric analysis of *U2af1*^S34F/+^ PB and BM demonstrated significant reductions in both myeloid and lymphoid lineages (Supplementary Fig. 1E, F). In contrast, *U2af1*^Q157R/+^ mice had no significant PB or BM changes except for elevated MCV (Fig. 1F and Supplementary Fig. 1E, F). Assessment of BM hematopoietic stem and progenitor cells (HSPC) four weeks after pIpC treatment revealed that *U2af1*^S34F/+^ mice had significantly reduced numbers of short-term hematopoietic stem cell (ST-HSC), KL, and common myeloid progenitor (CMP) populations with increased numbers of multipotent progenitor (MPP)2 and MPP3 populations compared with control mice (Fig. 1G). *U2af1*^Q157R/+^ mice also had significantly reduced numbers of ST-HSC and KL populations and non-significant reductions in both CMP and megakaryocyte-erythroid progenitor (MEP) cells compared with control mice (Fig. 1G). *U2af1*^S34F/+^ mice had a significant block in erythroid development in the BM and spleen, with an increased proportion of immunophenotypically defined nucleated erythroblasts (Ter119^lo/hi^CD71^hi^) and a decreased proportion of enucleated erythrocytes (Ter119^hi^CD71^lo^). In contrast, *U2af1*^Q157R/+^ mice had a smaller but non-significant increase in Ter119^hi^CD71^hi^ cells in the spleen (Supplementary Fig. 1G–I).

**Fig. 2:**
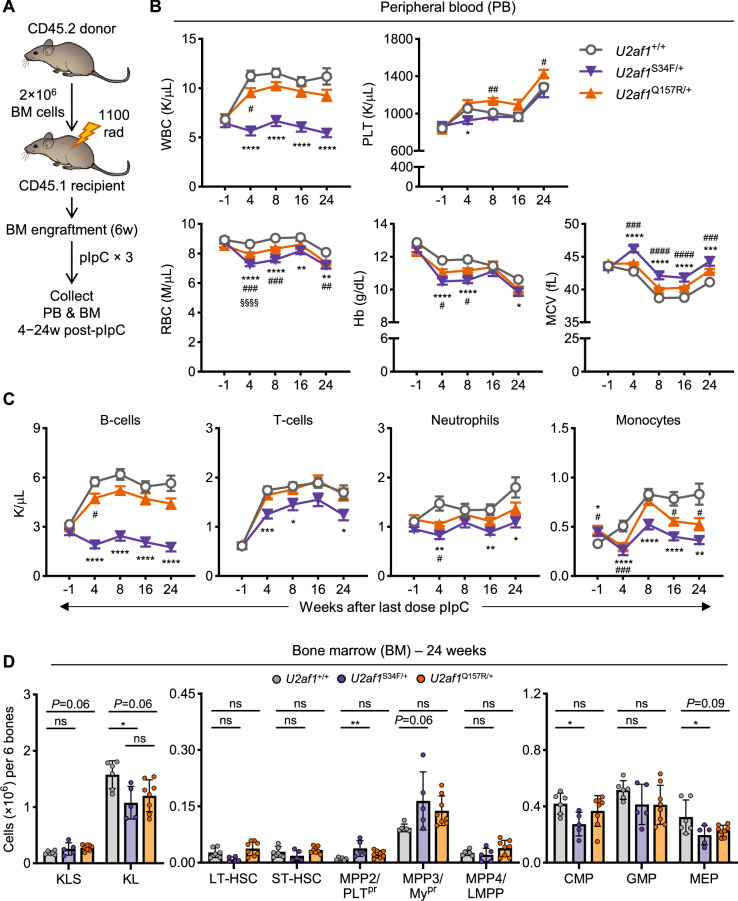
U2AF1^S34F^ and U2AF1^Q157R^ cause different cell-intrinsic effects on hematopoiesis. **A** Transplant assay design. CD45.2^+^ donor BM cells from *U2af1*^+/+^, *U2af1*^S34F/+^, or *U2af1*^Q157R/+^ mice (all *Mx1*-*Cre*^+^) were transplanted into lethally irradiated WT congenic (CD45.1^+^) recipient mice. Recipient mice were treated with pIpC at 6 weeks post-transplant. **B** Complete blood counts of PB samples from recipient mice before (−1 week) and up to 24 weeks post-pIpC. **C** Flow cytometric analysis of PB samples was performed before and after pIpC to determine absolute counts of lymphoid (B-cells or T-cells) and myeloid (Neutrophils or Monocytes) cells. For (**B**, **C**), *N* = 28–30 recipient mice per genotype, pooled from two independent experiments. **D** Absolute cell counts of BM HSPC populations in recipient mice were determined by flow cytometric analysis at 24 weeks post-pIpC. *N* = 5–8 recipient mice per genotype, pooled from two independent experiments. See also Supplementary Fig. 2. Results represent the mean ± standard error of the mean (SEM) (**B**, **C**) or mean ± SD (**D**). A mixed effects analysis with repeated measures and Tukey multiple comparison correction (**B**, **C**) or one-way ANOVA with Tukey multiple comparison correction (**D**) was used for the comparison of groups. *P < 0.05; **P < 0.01; ***P < 0.001; ****P < 0.0001. ns, not significant (or labeled if *P* < 0.10). Symbols (*U2af1*^+/+^ vs *U2af1*^S34F/+^ [*]; *U2af1*^+/+^ vs *U2af1*^Q157R/+^ [#]; *U2af1*^S34F/+^ vs *U2af1*^Q157R/+^ [§]) are used to differentiate comparisons in (**B**, **C**).

To better evaluate the cell-intrinsic effects of both mutants on hematopoiesis, we transplanted BM from *U2af1*^Q157R/+^, *U2af1*^S34F/+^, or *U2af1*^+/+^ control mice (CD45.2^+^; all *Mx1-Cre*^+^) into lethally irradiated WT congenic (CD45.1^+^) recipient mice (average CD45.2^+^ chimerism was >85% at 6 weeks and >88% at 24 weeks post-pIpC for all genotypes). Following engraftment, we treated mice (including controls) with pIpC to induce expression of S34F and Q157R in donor-derived cells (Fig. 2A). Four weeks after pIpC treatment, PB (Fig. 2B, C) and BM changes (Supplementary Fig. 2A, C) reflected similar overall trends observed in native hematopoiesis (Fig. 1F and Supplementary Fig. 2E) for both mutant mice. At 24 weeks, both mutant mice had significantly reduced PB RBC counts with increased MCV, as well as decreased hemoglobin in *U2af1*^S34F/+^ mice. *U2af1*^Q157R/+^ mice also had mildly increased platelet counts (Fig. 2B). *U2af1*^S34F/+^ mice had significantly reduced PB and BM myeloid and lymphoid lineage cells, while *U2af1*^Q157R/+^ mice had significantly decreased PB monocytes and a non-significant increase in BM monocytes (Fig. 2C and Supplementary Fig. 2D). Although myeloid and lymphoid lineages were significantly decreased in *U2af1*^S34F/+^ mouse spleens at 4 weeks, there were no significant changes at 24 weeks (Supplementary Fig. 2B, E). HSPC populations reflected similar significant overall trends at 24 weeks compared to 4 weeks for *U2af1*^S34F/+^ mice (Fig. 2D and Supplementary Fig. 2C). *U2af1*^Q157R/+^ mice also showed similar, but non-significant, trends in HSPC population numbers at 24 weeks compared to 4 weeks (Fig. 2D and Supplementary Fig. 2C). A similar erythroid differentiation block was observed in the spleen at 24 weeks compared to 4 weeks (Supplementary Figs. 1H, I and 2F, G).

### *U2af1*^S34F/+^ HSCs are significantly more impaired than *U2af1*^Q157R/+^ HSCs in BM repopulation assays

**Fig. 3:**
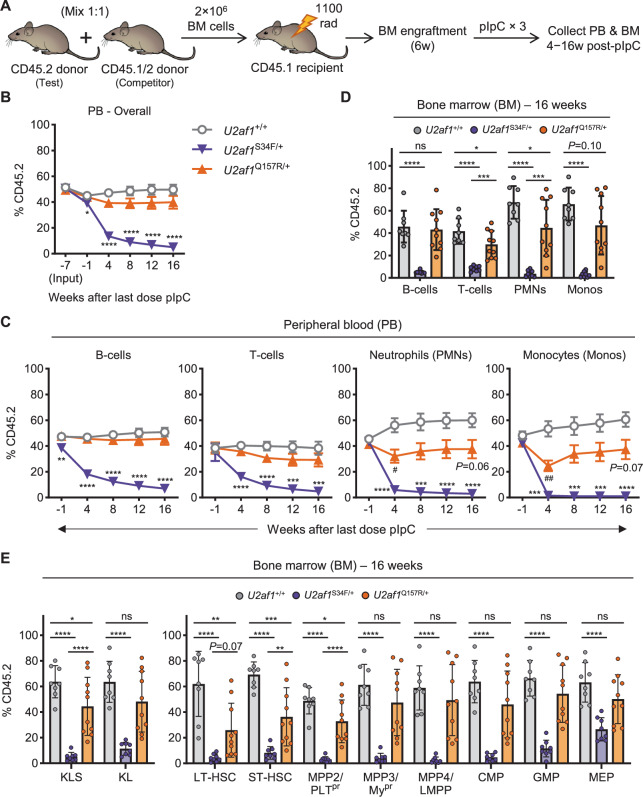
*U2af1*^S34F/+^ HSCs are significantly more impaired than *U2af1*^Q157R/+^ HSCs in BM repopulation assays. **A** Competitive transplant assay design. CD45.2^+^ (test) donor BM cells from *U2af1*^+/+^, *U2af1*^S34F/+^, or *U2af1*^Q157R/+^ mice (all *Mx1*-*Cre*^+^) were each mixed 1:1 with CD45.1^+^/CD45.2^+^ competitor BM cells and transplanted into lethally irradiated WT congenic (CD45.1^+^) recipient mice. Recipient mice were treated with pIpC at 6 weeks post-transplant. **B**, **C** Donor cell chimerism (CD45.2^+^) was assessed on overall PB leukocytes (**B**) and lymphoid (B-cells or T-cells) and myeloid (Neutrophils or Monocytes) cell populations (**C**) from recipient mice before (−1 week) and up to 16 weeks post-pIpC. Input (−7 weeks) refers to the 1:1 BM cell mixtures transplanted into recipient mice. **D**, **E** Donor cell chimerism (CD45.2^+^) was assessed on BM lymphoid (B-cells or T-cells) and myeloid (PMNs or Monos) cell populations (**D**) and BM HSPC populations (**E**) from recipient mice at 16 weeks post-pIpC. *N* = 8-10 recipient mice per genotype pooled from two independent experiments (**B**–**E**). See also Supplementary Fig. 3. Results represent the mean ± SEM (**B**, **C**) or mean ± SD (**D**, **E**). A two-way ANOVA with repeated measures and Tukey multiple comparison correction (**B**, **C**) or one-way ANOVA with Tukey multiple comparison correction (**D**, **E**) were used for the comparison of groups. *P < 0.05; **P < 0.01; ***P < 0.001; ****P < 0.0001. ns, not significant (or labeled if *P* < 0.10). Symbols (*U2af1*^+/+^ vs *U2af1*^S34F/+^ [*]; *U2af1*^+/+^ vs *U2af1*^Q157R/+^ [#]) are used to differentiate comparisons in (**B**, **C**).

To compare the effects of S34F or Q157R expression on HSC reconstitution capacity, we performed competitive BM transplantation experiments. Lethally irradiated WT congenic recipient mice (CD45.1^+^) were transplanted with whole BM “test” cells from *U2af1*^Q157R/+^, *U2af1*^S34F/+^, or *U2af1*^+/+^ control mice (CD45.2^+^; all *Mx1-Cre*^+^) mixed with an equal number of competitor BM cells from WT congenic mice (CD45.1^+^/CD45.2^+^). Following engraftment, we treated mice (including controls) with pIpC to induce expression of S34F and Q157R in donor-derived cells (Fig. 3A). Consistent with previous characterization, [19] we observed significant multi-lineage reductions in PB, BM, and spleen donor cell chimerism (CD45.2^+^) for *U2af1*^S34F/+^ compared to *U2af1*^+/+^ test cells (Fig. 3B–D and Supplementary Fig. 3A). In contrast, the reduction in overall and multilineage PB donor cell chimerism for *U2af1*^Q157R/+^ compared to *U2af1*^+/+^ test cells was less severe relative to *U2af1*^S34F/+^ test cells (Fig. 3B, C). In addition, there were variable reductions in donor cell chimerism of PB, BM, and spleen myeloid lineages for *U2af1*^Q157R/+^ compared to *U2af1*^+/+^ test cells (Fig. 3C, D and Supplementary Fig. 3A). Donor cell chimerism for all BM HSPC populations were significantly reduced for *U2af1*^S34F/+^ compared to *U2af1*^+/+^ test cells (Fig. 3E). However, reduced *U2af1*^Q157R/+^ donor cell chimerism was restricted to the HSC and MPP2 populations, but not to the same degree as for *U2af1*^S34F/+^ (Fig. 3E). As observed in primary transplants, *U2af1*^S34F/+^ HSPC and mature lineage cells are nearly absent in the BM and PB of secondary transplant recipients (Supplementary Fig. 3B–D). BM and PB donor cell chimerism were unchanged or modestly reduced for *U2af1*^Q157R/+^ HSPC and mature lineage cells following secondary transplant compared to chimerism values in primary transplant animals (Supplementary Fig. 3B–D). These results further suggest that *U2af1*^Q157R/+^ HSC are less functionally compromised than *U2af1*^S34F/+^ HSC. Since pIpC has been reported to have effects on HSC quiescence, proliferation, and stress responses, we also performed an additional competitive transplant study using isogenic transgenic mice that express a doxycycline-inducible single copy *U2AF1*^S34F^ or *U2AF1*^WT^ transgene from the *Col1a1* locus [21]. Consistent with previous characterization [21], S34F mutant HSPCs have a competitive disadvantage compared to WT HSPCs in the absence of inflammation induced by pIpC, and the reduction in chimerism is dose dependent, further supporting that the effects are cell intrinsic (Supplementary Fig. 3E).

### Hemizygous *U2af1*^Q157R/−^ and *U2af1*^S34F/−^ HSCs are both severely impaired in BM repopulation assays

**Fig. 4:**
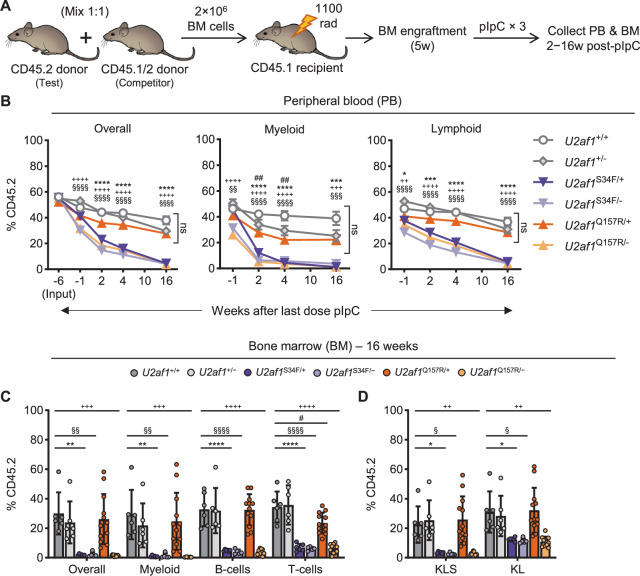
Hemizygous *U2af1*^Q157R/−^ and *U2af1*^S34F/−^ HSCs are severely impaired in BM repopulation assays. **A** Competitive transplant assay design. CD45.2^+^ (test) donor BM cells from *U2af1*^+/+^, *U2af1*^+/−^, *U2af1*^S34F/+^, *U2af1*^Q157R/+^, *U2af1*^S34F/−^, or *U2af1*^Q157R/−^ mice (all *Mx1-Cre*^+^) were each mixed 1:1 with CD45.1^+^/CD45.2^+^ competitor BM cells and transplanted into lethally irradiated WT congenic (CD45.1^+^) recipient mice. Recipient mice were treated with pIpC at 5 weeks post-transplant. **B** Donor cell chimerism (CD45.2^+^) was assessed on PB from recipient mice before (−1 week) and up to 16 weeks post-pIpC. Input (−6 weeks) refers to the 1:1 BM cell mixtures transplanted into recipient mice. Overall, Myeloid (CD11b^+^ cells), and Lymphoid (B-cells and T-cells) PB chimerism are shown. **C**, **D** Donor cell chimerism (CD45.2^+^) was assessed on BM myeloid (CD11b^+^ cells) and lymphoid (B-cells or T-cells) cell populations (**C**) and BM HSPC populations (**D**) from recipient mice at 16 weeks post-pIpC. For (**B**–**D**), data are from a single experiment in which a pool of competitor BM cells (*N* = 3 donors) was individually mixed with test BM cells from *N* = 15 different donors (*N* = 2–4 per genotype) prior to transplant into *N* = 80 recipients (*N* = 5-8 recipient mice per BM cell mixture and *N* = 10–20 total recipient mice per genotype group). BM analysis was performed on a subset (*N* = 6–12 randomized mice) of each genotype group. Data from one *U2af1*^S34F/−^ mouse was identified as a significant outlier (Grubb’s test, *P* < 0.05) and removed from final analysis. See also Supplementary Fig. 4. Results represent the mean ± SEM (**B**) or mean ± SD (**C**, **D**). A two-way ANOVA with repeated measures and Tukey multiple comparison correction (**B**) or one-way ANOVA with Tukey multiple comparison correction (**C**, **D**) were used for the comparison of groups. *P < 0.05; **P < 0.01; ***P < 0.001; ****P < 0.0001. ns, not significant (or labeled if *P* < 0.10). Symbols (*U2af1*^+/+^ vs *U2af1*^S34F/+^ [*]; *U2af1*^+/+^ vs *U2af1*^Q157R/+^ [#]; *U2af1*^+/+^ vs *U2af1*^S34F/−^ [§]; *U2af1*^+/+^ vs *U2af1*^Q157R/−^ [+]) are used to differentiate comparisons in (**B**–**D**).

We previously demonstrated that cell survival and reconstitution capacity are severely reduced for HSCs that express mutant U2AF1^S34F^ without WT U2AF1 expression (hemizygous *U2af1*^S34F/−^) [20]. Given the mild reconstitution defect observed for *U2af1*^Q157R/+^ cells, we hypothesized that mutant U2AF1^Q157R^ cells may not require the expression of WT U2AF1 for cell survival. To test this, we performed competitive BM transplantation experiments using test cells from three additional genotypes of mice: *U2af1*^Q157R/−^, *U2af1*^S34F/−^, and *U2af1*^+/−^ mice (all *Mx1-Cre*^+^; Fig. 4A). Hemizygous conditional knock-in mice were generated by crossing heterozygous floxed mutant (S34F or Q157R) mice to heterozygous floxed knockout mice. Consistent with previous characterization [20], we noted a rapid and significant loss in mature cell and HSPC donor cell chimerism (CD45.2^+^) in the PB, BM, and spleen for hemizygous *U2af1*^S34F/−^ (but not *U2af1*^+/−^) compared to *U2af1*^+/+^ test cells following administration of pIpC (Fig. 4B–D and Supplementary Fig. 4A, B). We also observed an identical rapid loss in mature cell and HSPC chimerism for hemizygous *U2af1*^Q157R/−^ compared to *U2af1*^+/+^ test cells (Fig. 4B–D and Supplementary Fig. 4A, B). This indicates that the expression of WT U2AF1 is required for the viability of either U2AF1^S34F^ or U2AF1^Q157R^ mutant expressing HSCs, consistent with *U2AF1* being a haplo-essential gene [20] and reinforcing that the *U2af1*^Q157R^ allele impairs U2AF1 function despite the less severe phenotype compared to *U2af1*^S34F^.

### U2AF1^S34F^ and U2AF1^Q157R^ induce distinct gene expression changes in mouse myeloid progenitor cells

**Fig. 5:**
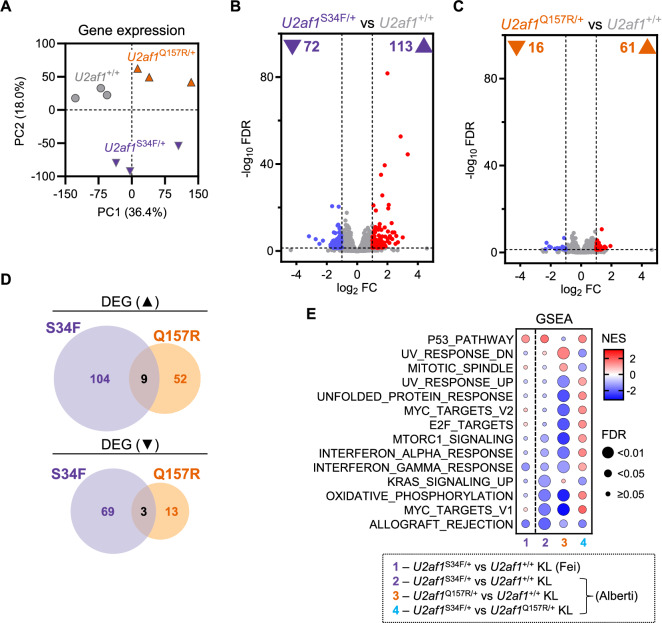
U2AF1^S34F^ and U2AF1^Q157R^ induce distinct gene expression changes in myeloid progenitor cells. Assessment of differential gene expression by RNA-seq in BM KL cells from *U2af1*^+/+^, *U2af1*^S34F/+^, and *U2af1*^Q157R/+^ mice under native hematopoiesis conditions (as in Fig. 1D). *N* = 3 KL cell samples per genotype. **A** Unsupervised principal component (PC) analysis of gene expression levels in KL cells. Volcano plot of differentially expressed genes (DEG; FDR < 0.05 and |log_2_ FC| > 1 vs *U2af1*^+/+^) in KL cells from *U2af1*^S34F/+^ (**B**) or *U2af1*^Q157R/+^ (**C**) mice. The numbers of up- (▲) and down- (▼) regulated DEG are listed. **D** Overlap of upregulated (top) and downregulated (bottom) DEG in *U2af1*^S34F/+^ and *U2af1*^Q157R/+^ KL cells. **E** Gene set enrichment analysis (GSEA) for Hallmark gene sets that were significantly enriched (FDR < 0.05) in *U2af1*^S34F/+^ vs *U2af1*^Q157R/+^ KL cells (column 4). Normalized enrichment scores (NES) for *U2af1*^S34F/+^ vs *U2af1*^+/+^ (column 2) and *U2af1*^Q157R/+^ vs *U2af1*^+/+^ (column 3) KL cells are also shown. Reanalyzed RNA-seq data (GSE112174) from *U2af1*^+/+^ and *U2af1*^S34F/+^ KL cells under native hematopoiesis conditions in Fei et al. [19] (column 1) is also included. Circle color indicates the NES score for each term, and size is proportional to the magnitude of the FDR (q-value).

To characterize the effects of mutant U2AF1 on gene expression in vivo, we performed RNA-seq of total RNA (rRNA-depleted) from BM myeloid progenitor (KL) cells from *U2af1*^Q157R/+^, *U2af1*^S34F/+^, or *U2af1*^+/+^ control mice (all *Mx1-Cre*^+^) under native hematopoiesis conditions (as in Fig. 1D). KL cells were isolated by FACS at 4 weeks after completion of pIpC injections and the variant allele frequencies of the S34F and Q157R mutations were near 50% (Supplementary Fig. 5A). Unsupervised principal component analysis of gene expression values (*N* = 19312 genes) segregated *U2af1*^Q157R/+^, *U2af1*^S34F/+^, and *U2af1*^+/+^ KL cells (Fig. 5A and Supplementary Table 3). Reanalysis of *U2af1*^S34F/+^ Native KL RNA-seq data published by Fei et al. [19] demonstrated a strong concordance in gene expression changes with our *U2af1*^S34F/+^ KL data (Supplementary Fig. 5B, C). In our dataset, we identified 185 differentially expressed genes (DEGs; FDR < 0.05 and |log_2_FC| > 1) in *U2af1*^S34F/+^ compared to *U2af1*^+/+^ control mice (Fig. 5B) and 77 DEGs in *U2af1*^Q157R/+^ KL cells (Fig. 5C). There were only 12 DEGs shared between *U2af1*^S34F/+^ and *U2af1*^Q157R/+^ KL cells (4.8%; Fig. 5D) with no overlap in gene ontology (GO) analysis (Supplementary Fig. 5D, E and Supplementary Table 4). Gene set enrichment analysis (GSEA) revealed significant positive enrichment of the p53 pathway in *U2af1*^S34F/+^ KL cells and negative enrichment of immune response-related Hallmark pathways in both *U2af1*^S34F/+^ and *U2af1*^Q157R/+^ KL cells compared to *U2af1*^+/+^ KL cells (Fig. 5E).

### U2AF1^S34F^ and U2AF1^Q157R^ induce distinct alternative pre-mRNA splicing changes in myeloid progenitor cells

**Fig. 6:**
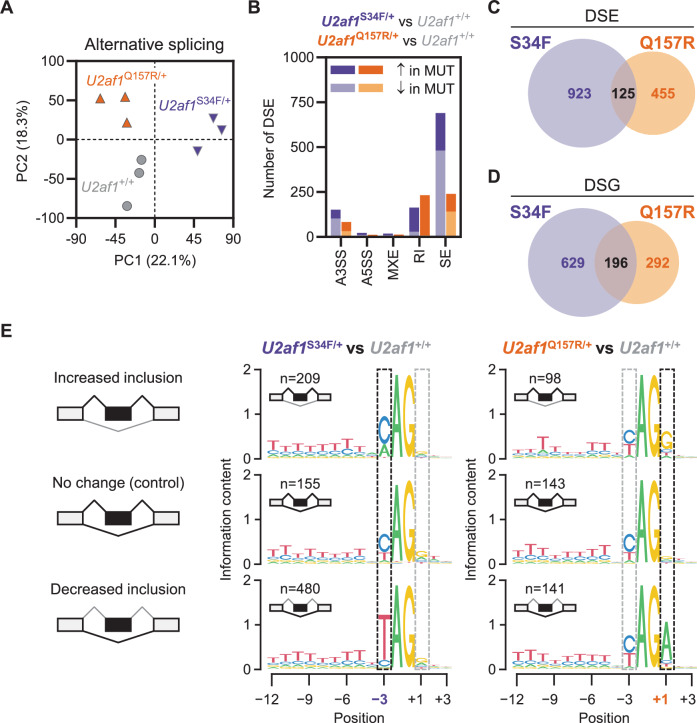
U2AF1^S34F^ and U2AF1^Q157R^ induce distinct alternative pre-mRNA splicing changes in myeloid progenitor cells. Assessment of differential alternative pre-mRNA splicing by RNA-seq in BM KL cells from *U2af1*^+/+^, *U2af1*^S34F/+^, and *U2af1*^Q157R/+^ mice under native hematopoiesis conditions (Fig. 1D). *N* = 3 KL cell samples per genotype. **A** Unsupervised principal component (PC) analysis of exon-inclusion ratios (referred to as “percent spliced-in” or “PSI”) for all annotated alternative splicing events in KL cells. **B** Number and type (alternative 3’ or 5’ splice sites [A3SS, A5SS], mutually exclusive exons [MXE], retained introns [RI], and skipped exons [SE]) of differentially spliced events (DSE; FDR < 0.05 and |ΔPSI| > 0.05 vs *U2af1*^+/+^) in KL cells from *U2af1*^S34F/+^ (left bars) or *U2af1*^Q157R/+^ (right bars) mice. **C** Overlap of DSE in *U2af1*^S34F/+^ and *U2af1*^Q157R/+^ KL cells. **D** Overlap of differentially spliced genes (DSG) in *U2af1*^S34F/+^ and *U2af1*^Q157R/+^ KL cells. DSE from (**C**) were converted to DSG for analysis. **E** Analysis of consensus 3’ splice site (3’SS) sequences from control (i.e., no change in mutant vs *U2af1*^+/+^) and differentially spliced SE events in *U2af1*^S34F/+^ (middle) or *U2af1*^Q157R/+^ (right) KL cells. The highlighted −3 and +1 positions of the 3’SS recapitulate the aberrant consensus 3’SS sequence dependencies identified previously in *U2AF1*^S34F^ and *U2AF1*^Q157R^ MDS patients. See also Supplementary Fig. 6.

Using the same bulk RNA-seq data, we next characterized the effects of mutant U2AF1 on alternative mRNA splicing in vivo. We employed replicate multivariate analysis of transcript splicing (rMATS) [24] to assess differential alternative pre-mRNA splicing of five different types of annotated splicing events (alternative 3’ or 5’ splice sites [A3SS, A5SS], mutually exclusive exons [MXE], retained introns [RI], and skipped exons [SE]) in *U2af1*^Q157R/+^, *U2af1*^S34F/+^, and *U2af1*^+/+^ KL cells. Unsupervised principal component analysis of inclusion ratios (referred to as “percent spliced-in” or “PSI”) for all annotated alternative splicing events (*N* = 11580) revealed that global alternative pre-mRNA splicing is distinct between *U2af1*^Q157R/+^, *U2af1*^S34F/+^, and *U2af1*^+/+^ KL cells (Fig. 6A and Supplementary Tables 5–7).

We then applied rMATS to identify 1048 and 580 differentially spliced events (DSEs; FDR < 0.05 and |ΔPSI| > 0.05 vs *U2af1*^+/+^) in *U2af1*^S34F/+^ and *U2af1*^Q157R/+^ KL cells, respectively (Fig. 6B and Supplementary Table 8). We also applied our rMATS analysis pipeline to the *U2af1*^S34F/+^ Native KL RNA-seq dataset published by Fei et al. [19] (Supplementary Fig. 6A, B) and observed a strong concordance (i.e., unidirectional ΔPSI values) between DSEs shared between the two *U2af1*^S34F/+^ KL datasets (Supplementary Fig. 6C). Thus, rMATS analysis of independent RNA-seq data demonstrates that the *U2af1*^S34F/+^ mouse model produces robust and reproducible gene expression and alternative pre-mRNA splicing changes in hematopoietic cells in vivo (Supplementary Figs. 5B and 6C). In line with previous studies of U2AF1 mutant cell lines and patient HSPC, SE events represented the majority of DSEs identified in U2AF1 mutant mouse KL cells (Fig. 6B and Supplementary Fig. 6A) [13, 14, 19, 25]. *U2af1*^S34F/+^ SE DSEs also favored exon exclusion (“skipping”) over exon inclusion [14]. Of note, *U2af1*^Q157R/+^ DSEs were more equally distributed between RI and SE events (Fig. 6B). The overlap of DSE shared between *U2af1*^S34F/+^ and *U2af1*^Q157R/+^ KL cells was low (125 events or 8.3%; Fig. 6C). Conversion of DSE to differentially spliced genes (DSG) revealed 196 genes (17.5%) aberrantly spliced in common between the two mutants (Fig. 6D). GO analysis revealed that DSGs from *U2af1*^S34F/+^ KL cells were most significantly enriched in mRNA binding and metabolism gene sets, as well as histone post-translational modification and stress granule [14] related gene sets (Supplementary Fig. 6D and Supplementary Table 9). DSGs from *U2af1*^Q157R/+^ KL cells were enriched in mRNA gene sets to a weaker extent than *U2af1*^S34F/+^ (Supplementary Fig. 6D and Supplementary Table 9).

Analysis of consensus 3’ splice site (3’SS) sequences from differentially spliced SE events in *U2af1*^S34F/+^ and *U2af1*^Q157R/+^ KL cells confirmed previous dependencies identified in U2AF1 mutant cell lines and patient HSPC [11, 13, 14, 19, 21, 23, 25–27]. Specifically, exon inclusion favored a C and exon exclusion favored a T at the −3 position of the 3’SS in *U2af1*^S34F/+^ cells (Fig. 6E, middle). In contrast, exon inclusion favored a G and exon exclusion favored an A at the +1 position of the 3’SS in *U2af1*^Q157R/+^ cells (Fig. 6E, right). Overall, these findings highlight that the U2AF1^S34F^ and U2AF1^Q157R^ mutants induce significant but distinct changes to alternative mRNA splicing in vivo.

### *U2af1*^S34F/+^ and *U2af1*^Q157R/+^ mouse models recapitulate alternative pre-mRNA splicing changes found in MDS and AML patients

**Fig. 7:**
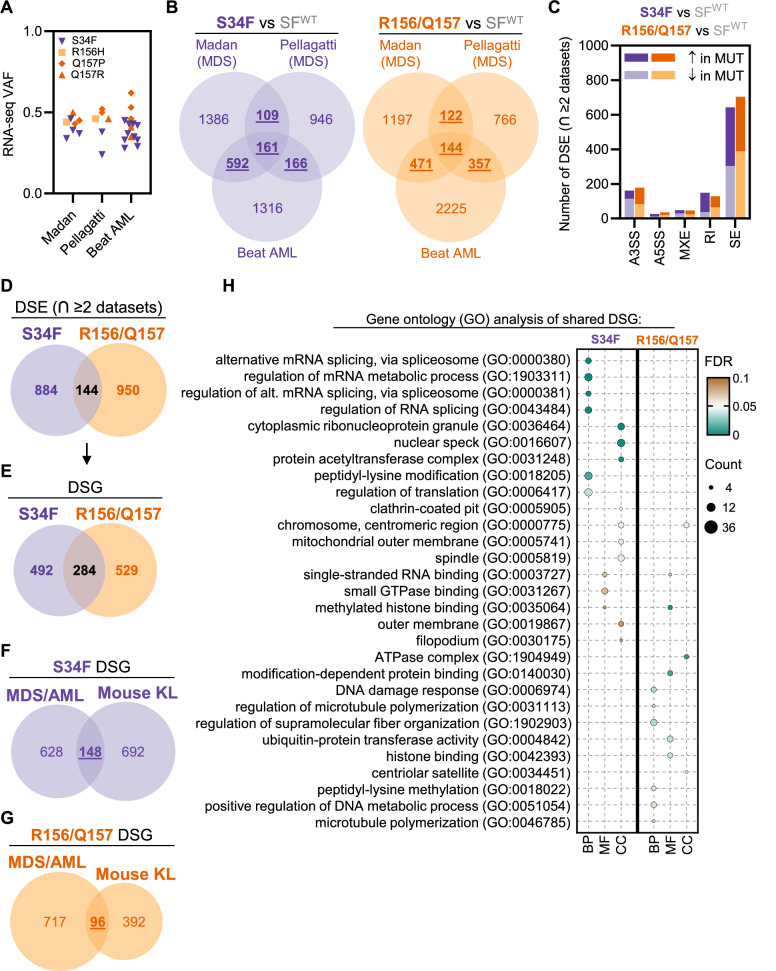

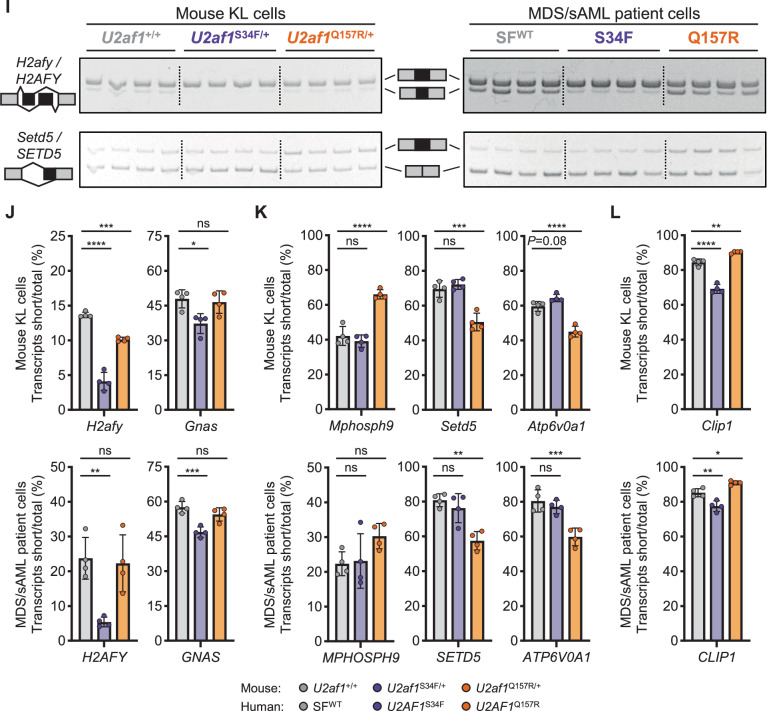
*U2af1*^S34F/+^ and *U2af1*^Q157R/+^ mouse models recapitulate alternative pre-mRNA splicing changes found in MDS and AML patients. Assessment of differential alternative pre-mRNA splicing in BM cells from splicing factor WT [SF^WT^] MDS and AML patients and those harboring *U2AF1*^S34F^ (S34F) or *U2AF1*^R156H/Q157(P/R)^ (R156/Q157) mutations in three publicly available RNA-seq datasets (Madan et al., [12] Pellagatti et al., [11] and Beat AML [28]). RNA-seq data (GSE128429, GSE114922, and phs001657.v1.p1) were reanalyzed for this study. *N* = 2–10 samples per mutant genotype per study. *N* = 8 (Madan), 40 (Pellagatti), or 279 (Beat AML) SF^WT^ samples. **A** BM cell variant allele frequencies (VAF) of S34F, R156H, and Q157(P/R) mutations in *U2AF1* mRNA from MDS and AML patients harboring *U2AF1* mutations in Madan, Pellagatti, and Beat AML. **B** Total number and intersection of differentially spliced events (DSE; FDR < 0.05 and |ΔPSI| > 0.05 vs SF^WT^ patients) in BM cells from MDS and AML patients harboring S34F or R156/Q157 mutations in Madan, Pellagatti, and Beat AML. See also Supplementary Fig. 7. DSE shared (∩) between at least two datasets are underlined and bolded. **C** Number and type (alternative 3’ or 5’ splice sites [A3SS, A5SS], mutually exclusive exons [MXE], retained introns [RI], and skipped exons [SE]) of DSE (∩ ≥ 2 MDS/AML datasets) in BM cells from patients harboring S34F (left bars) or R156/Q157 (right bars) mutations. **D** Overlap of DSE (∩ ≥ 2 MDS/AML datasets) in BM cells from MDS and AML patients harboring S34F or R156/Q157 mutations. **E** Overlap of differentially spliced genes (DSG) in BM cells from MDS and AML patients harboring S34F or R156/Q157 mutations. DSE from (**D**) were converted to DSG for analysis. **F**, **G** Overlap of DSG from (**E**) (MDS-AML) with DSG from Fig. 6D (Mouse KL) for S34F (**F**) or R156/Q157 (**G**) mutations. **H** GO analysis of S34F (left) and R156/Q157 (right) shared DSGs from (**F**, **G**). Circle size is proportional to the gene count for each term, and the color indicates the magnitude of the FDR (q-value). REVIGO was used to consolidate 51 (S34F) or 40 (R156/Q157) gene sets into a representative subset of GO terms with gene counts ≥ 4 [43]. All significant GO terms are listed in Supplementary Table 17. **I**–**L** RT-PCR orthogonal confirmation of S34F or Q157R aberrantly spliced transcripts in mutant mouse KL (4 weeks post-pIpC) and MDS/s-AML patient cells. **I** Representative RT-PCR/polyacrylamide gel results for *H2afy*/*H2AFY* (aberrantly spliced by S34F, left) and *Setd5*/*SETD5* (aberrantly spliced by Q157R, right) prior to gel densitometry quantification. *N* = 4 samples per genotype. Quantification of aberrantly spliced transcripts in S34F (**J**), Q157P/R (**K**), or both (**L**). Results represent the mean ± SD (**J**–**L**). A one-way ANOVA with Tukey multiple comparison correction (**J**–**L**) was used for the comparison of groups. *P < 0.05; **P < 0.01; ***P < 0.001; ****P < 0.0001. ns, not significant (or labeled if *P* < 0.10).

To assess how well alternative splicing changes in mouse KL cells recapitulate changes seen in MDS and AML patient hematopoietic cells, we performed a meta-analysis using publicly available RNA-seq data from three published studies [11, 12, 28]. Each study included 2-10 U2AF1^S34F^ and only 1-2 U2AF1^Q157R^ patients. Therefore, U2AF1^R156H^ and U2AF1^Q157(P/R)^ patient samples were grouped together (*N* = 4–5 *U2AF1*^R156H/Q157(P/R)^ patients per study; Fig. 7A), consistent with previous studies demonstrating similar 3’SS sequence dependencies [13, 23, 25]. In each study, samples from MDS/AML patients who did not have identifiable mutations in *SF3B1* or *SRSF2* were used as a comparator (Splicing Factor [SF]^WT^). To allow for a more rigorous analysis of differential splicing, we reanalyzed the FASTQ files for each study using the same analysis workflows and applied the same significance thresholds (FDR < 0.05 and |ΔPSI| > 0.05 vs SF^WT^) as used for the analysis of mouse KL cells. Using this approach, we credentialed each of the three datasets (referred to as Madan, [12] Pellagatti, [11] and Beat AML [28]) (Fig. 7A and Supplementary Fig. 7A–I and Supplementary Tables 10–15). Specifically, SE events were the most frequent DSE type identified in each study for S34F and R156/Q157 (Supplementary Fig. 7A–C) and these events favored the characteristic consensus 3’SS sequence dependencies identified previously (Supplementary Fig. 7G–I) [11, 13, 14, 19, 21, 23, 25–27]. To increase the rigor of our meta-analysis we prioritized only the DSEs that were shared between at least two of the three MDS/AML datasets for either *U2AF1*^S34F^ or *U2AF1*^R156/Q157^ (Fig. 7B and Supplementary Table 16). The distribution of these DSEs was similar to each individual dataset, with SE events still representing the majority event type in U2AF1 mutant MDS/AML cells (Fig. 7C). As in the mice, the overlap of DSE shared between *U2AF1*^S34F^ and *U2AF1*^R156/Q157^ MDS/AML cells was low (144 of 1978 events or 7.3%; Fig. 7D). Conversion of DSE to DSG revealed a total of 284 of 1305 genes (21.8%) aberrantly spliced in common between the two mutants (Fig. 7E).

The overlap of DSG identified in human and mouse cells revealed that approximately 20% of aberrantly spliced genes in KL (mouse) cells were also mis-spliced in MDS/AML (human) cells for both U2AF1^S34F^ (17.6% shared) and U2AF1^Q157R^ (19.7% shared) mutants (Fig. 7F, G). *EZH2* was not consistently mis-spliced in both human and mouse samples for either *U2AF1* hotspot mutation. GO analysis revealed that shared S34F DSGs were most significantly enriched in mRNA binding and metabolism gene sets, as well as stress granule [14] and mRNA translation related gene sets (Fig. 7H and Supplementary Table 17). Shared Q157R DSGs were less significantly enriched in mRNA gene sets than S34F. Histone binding and DNA damage response gene sets were among some of the significantly enriched gene sets for Q157R DSGs (Fig. 7H and Supplementary Table 17).

We validated several of these putatively shared aberrant splicing changes identified by rMATS analysis by performing RT-PCR followed by gel electrophoresis of RNA isolated from additional mouse KL cell samples (*N* = 4 per genotype; 4 weeks post-pIpC treatment) and MDS patient samples (*N* = 4–9 per genotype). Consistent with previous observations [19, 21, 27, 29, 30], we confirmed aberrant splicing of functionally relevant transcripts (*H2AFY* and *GNAS*) in U2AF1^S34F^ mutant mouse KL and MDS cells (Fig. 7I, J). We also demonstrate that aberrantly spliced transcripts (*MPHOSPH9*, *SETD5*, *ATP6V0A1*, and *CLIP1*) in U2AF1^Q157R^ mutant MDS patient cells are similarly mis-spliced in KL cells from *U2af1*^Q157R/+^ mice (Fig. 7I, K, L). Aberrant splicing of *CLIP1* is one example of an SE event that is differentially spliced in opposite directions by U2AF1^S34F^ (increased exon inclusion) and U2AF1^Q157R^ (increased exon skipping/exclusion) in mouse and human cells (Fig. 7L). Aberrant splicing of these same transcripts was also confirmed by RT-PCR followed by gel electrophoresis using RNA isolated from Kit^+^ BM cell samples from *U2af1*^S34F/+^ and *U2af1*^Q157R/+^ mice at 24 weeks post-pIpC treatment (Supplementary Fig. 7J–L), further highlighting the distinct and durable splicing differences induced by these two U2AF1 mutants.

### *U2AF1*^R156/Q157^ mutations are enriched in patients with CMML and MPN compared to *U2AF1*^S34F^ mutations

**Fig. 8:**
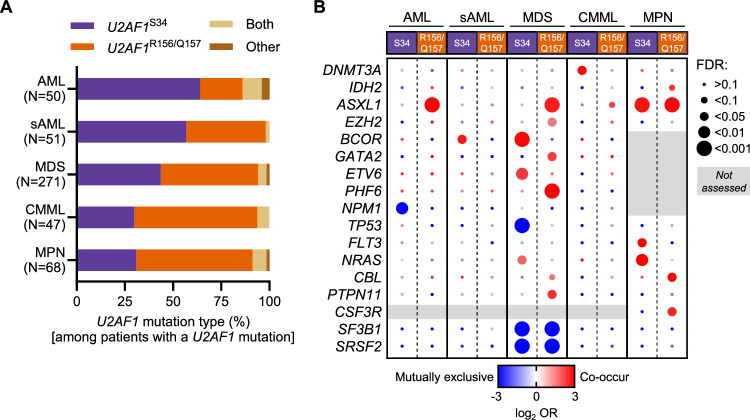
Frequency of *U2AF1*^S34^ and *U2AF1*^R156/Q157^ hotspot mutations and co-occurrence with other gene mutations differ in myeloid malignancies. **A** Frequency of *U2AF1* hotspot mutations in myeloid malignancy patients. Patients with a *U2AF1* mutation(s) (i.e., S34[F/Y], R156H/Q157[P/R], both S34 and R156/Q157, or “other” rare variants) and a diagnosis of AML (*N* = 50 patients), sAML (from MDS; *N* = 51 patients), MDS (*N* = 271 patients), CMML (*N* = 47 patients), and MPN (*N* = 68 patients), were identified from 21 published studies (see Supplementary Methods and Supplementary Table 18). **B** Analysis of *U2AF1* hotspot mutation co-occurrence and mutual exclusivity in myeloid malignancies. Mutation data for patients with a diagnosis of AML (*N* = 1857 patients), sAML (from MDS; *N* = 458 patients), MDS (*N* = 3159 patients), CMML (*N* = 430 patients), and MPN (*N* = 1551 patients) were included from 20 published studies that performed *U2AF1* sequencing and had patient-level mutation data available for a common set of 23 (MPN) or 31 (AML, sAML, MDS, and CMML) genes sequenced across all studies (see Supplementary Methods and Supplementary Table 19). cBioPortal was used for the co-occurrence and mutual exclusivity of genomic alteration analysis within each disease group using the default settings. Genes with significant interactions (FDR < 0.1) with *U2AF1* are shown (for complete analysis see Supplementary Fig. 8A, C–G and Supplementary Table 20). Circle color indicates the log_2_ odds ratio (OR) for each gene pair and size is proportional to the magnitude of the FDR (q-value). A similar analysis using *P*-values is presented in Supplementary Fig. 8B.

Given the differences in gene expression, splicing, and hematopoietic phenotypes induced by *U2af1*^S34F/+^ and *U2af1*^Q157R/+^ mutations in mice, we asked if the two hotspot mutations were differentially enriched in various myeloid neoplasms. We identified 487 patients with a diagnosis of AML, sAML (from MDS), MDS, CMML, or MPN who had a *U2AF1* mutation based on available sequencing data and calculated the proportion of patients with *U2AF1*^R156/Q157^ or *U2AF1*^S34^ mutations (see Supplementary Methods). We observed that *U2AF1*^R156/Q157^ mutations were more common in CMML and MPN patients, *U2AF1*^S34^ mutations were more common in sAML and AML patients, and a similar proportion of both mutations occurred in MDS (Fig. 8A and Supplementary Table 18). The co-occurrence of *U2AF1* and signaling gene mutations also differed across myeloid neoplasms, with *NRAS* and *FLT3* mutations being more common with S34 mutations and *CBL*, *PTPN11* and *CSF3R* mutations more common with R156/Q157 mutations. Similar to previous reports by our group and others, we also observed preferential co-occurrence of other gene mutations with *U2AF1*^R156/Q157^ (e.g., *ASXL1*) or *U2AF1*^S34F^ (e.g., *BCOR*) mutations in MDS patients (Fig. 8B and Supplementary Fig. 8 and Supplementary Tables 19–20) [17, 18, 31].

## Discussion

In this study, we characterized the in vivo consequences of expressing two myeloid neoplasm-associated hotspot mutations in *U2AF1* that code for S34F and Q157R substitutions. Our results indicate that the two mutations induce distinct hematopoietic phenotypes in mice, suggesting that the *U2af1*^S34F^ and *U2af1*^Q157R^ mutations should not be conflated as they may impact disease pathogenesis differently in patients. Mice expressing *U2af1*^S34F^ have a more severe reduction in their PB and BM cell counts, and reduced HSPCs repopulating ability, compared to mice expressing *U2af1*^Q157R^. The expression and splicing of the majority of target genes are unique between the mutations, in both mouse and human samples, potentially driving the phenotypic differences induced by the two mutations. The two mutations co-occur with different gene mutations and are not equally represented in various myeloid neoplasms, suggesting that multiple mechanisms are likely to drive the pathogenesis of *U2AF1* mutant myeloid diseases.

Our mouse model results add to the growing body of literature highlighting the paradigm that different hotspot mutations in a specific cancer gene can lead to distinct functional consequences and should, therefore, not necessarily be conflated. In one of the more well-studied examples, different *KRAS* hotspot mutations (e.g., G12, G13, Q61) lead to varying levels of KRAS activation through modulation of distinct biochemical properties of KRAS [32]. In turn, different *KRAS* hotspot mutations confer different prognostic value in various cancers (e.g., colorectal cancer) and are predictive of response to chemotherapy and/or targeted therapies [32]. Similarly, the prognostic significance of distinct *SF3B1* mutations can be different in MDS, including their impact on overall survival [33, 34]. With respect to *U2AF1*, a prognostic scoring model for MF (MIPSS70 + v2.0) now incorporates the negative impact of Q157 (but not S34) codon mutations [35]. U2AF1 S34 and Q157 codon-specific clinical characteristics have also been reported in MDS, including differences in age, marrow blast percentage, and blood count parameters, although results vary across studies [15, 17, 31, 36, 37]. Codon-specific differences in outcomes also vary between studies for S34 and Q157 mutations. While Bernard et al. did not observe S34 and Q157 codon-specific differences in survival, leukemic transformation, or leukemia-free survival for MDS patients [31], Badar et al. observed reduced survival for patients with high-risk myeloid neoplasms and Q157 compared to the S34 mutation [17]. Differences in cohort characteristics, sample size, co-mutation patterns [31, 38] and mutation VAF levels [37] could impact these analyses. Larger, prospective studies are needed to overcome these limitations and address whether the hematopoietic phenotypes observed in our preclinical mouse models exist in patients and are associated with clinical outcomes.

The context in which these mutations are studied (e.g., with or without common co-mutations; using young or aged mice) may play an important role in establishing faithful MDS mouse models [39, 40]. Ultimately, the downstream consequences of splicing factor gene mutations may converge on a common disease phenotype in fully transformed cells that is shared across *U2AF1* hotspot mutations and possibly other splicing factor gene mutations. This could include R-loop induction or DNA damage response; shared features induced across splicing factor gene mutations.

In line with findings from previous studies using human cell lines and MDS/AML patient samples [27, 30], *U2af1*^S34F/+^ mice recapitulate aberrant splicing of *H2AFY* and *GNAS* genes. We also demonstrate that *U2af1*^Q157R/+^ mice recapitulate similar splicing changes in MDS/AML patient samples that were identified through our meta-analysis. However, we did not observe aberrant inclusion of the *Ezh2* poison exon in *U2af1*^S34F^ mice, a mis-spliced event previously reported in S34 mutant patient samples [41] and confirmed in our S34 meta-analysis of publicly available RNA-seq data. Interestingly, the *EZH2* poison exon was also mis-spliced in one *U2AF1*^Q157^ patient cohort (Madan et al. [12]), and a different *Ezh2* event was significantly mis-spliced in *U2af1*^Q157R^ mice. While there are differences in splicing changes observed across human and mouse cells bearing *U2AF1* mutations, it is likely that the phenotype induced by splicing factor gene mutations involves the cooperation of multiple downstream target genes, not defined solely by the alternative splicing of *EZH2*. Further studies are needed to fully understand the functional impact of each *U2AF1* hotspot mutation and splicing differences in patients (and mice) and determine whether these differences confer consistent prognostic or therapeutic value across the spectrum of myeloid malignancies.

Enrichment of *U2AF1*^S34^ vs *U2AF1*^Q157^ mutations in different myeloid diseases suggests that mutations may contribute to the disease phenotype by differences in the target genes that they dysregulate and/or cooperating gene mutations. Identifying and validating the key target genes that are dysregulated and confer mutation-specific cellular phenotypes will require future in vivo functional studies. Additionally, based on differences in hotspot mutations in other cancers, the cellular “soil” that a S34 or Q157 mutation occurs likely also matters. In *U2AF1*-mutated solid tumors, particularly lung adenocarcinomas and endometrial cancers, S34 codon mutations are highly enriched compared to Q157 codon mutations [42]. This observation is not specific to *U2AF1*, as *SF3B1* R625 codon mutations are enriched in uveal and cutaneous melanomas, whereas K700 mutations are more common in breast cancer and chronic lymphoid leukemia specimens [42]. The subtle phenotype in the *U2af1*^Q157R/+^ mouse, including lack of severe cytopenias and possibly a slight increase in platelets, could make Q157R cells more permissive to transformation with an MPN-associated cooperating mutation (e.g., *CSF3R*), resulting in higher blood counts, something that will require future studies. In contrast, S34F induces cytopenias in mice and may contribute to cytopenias seen in MDS. In addition, S34 and Q157 do not cooperate with the same mutations in MDS (e.g., *BCOR* with S34 > Q157 and *ASXL1* with Q157 > S34) [31], and this could impact mutation-associated phenotypes in patients [15, 17, 36, 37]. *U2AF1* hotspot mutation phenotypes could also be influenced by the order of cooperating gene mutation acquisition (i.e., *U2AF1* mutation occurring before or after a cooperating gene mutation) or the presence of hematopoietic stressors, requiring future experiments.

Since pIpC has been reported to have effects on HSC quiescence, proliferation, and stress responses, we showed that the competitive disadvantage of U2AF1^S34F^ mutant cells occurs following an inflammatory (e.g., pIpC) or non-inflammatory (e.g., doxycycline) signal to induce expression in knock-in or transgenic mice, respectively. Future studies could explore the contribution of inflammation to multiple U2AF1^Q157R^ hematopoietic phenotypes using a non-inflammatory Cre model (e.g., tamoxifen-inducible Cre), a limitation of the current study. However, the consistency of the *Mx1*-*Cre*^+^*; U2af1*^Q157R^ knock-in phenotype across multiple experimental contexts (i.e., native hematopoiesis, non-competitive transplant, competitive primary and secondary transplant) provides reassurance even in the absence of a pIpC-free experiment.

Finally, our prior findings that heterozygous knock-out (*U2af1*^+/−^) cells do not phenocopy heterozygous S34F knock-in (*U2af1*^S34F/+^) cells suggest that the S34F mutation induces a neomorphic or change in function, rather than loss of function [20]. In addition, hemizygous knock-in (*U2af1*^S34F/−^) cells were not viable, indicating that U2AF1^S34F^ mutant hematopoietic cells require the expression of WT U2AF1 for cell survival in vivo, consistent with *U2AF1* being a haplo-essential gene. We now report the same finding for the Q157R mutation. While the change in function induced by the Q157R mutation is much more subtle than the effects of the S34F mutation, these results indicate that both the S34F and Q157R mutant U2AF1 proteins lack critical WT functions that are necessary for viability, further supporting that both mutations induce a change in function.

Collectively, our results support that *U2AF1*^S34F^ and *U2AF1*^Q157R^ mutations induce distinct hematopoietic, gene expression, and RNA splicing phenotypes in vivo. Larger population studies will be needed to determine if these phenotypic changes translate into clinico-pathologic differences in patients, warranting separate classification.

## Supplementary information

Table S1 List of primers

Table S2 Clinical characteristics of MDS and s-AML patients

Table S3 Mouse KL gene expression

Table S4 Gene ontology of mouse KL DEG

Table S5 Mouse KL S34F alternative splicing

Table S6 Mouse KL Q157R alternative splicing

Table S7 Mouse KL S34F_Fei alternative splicing

Table S8 Mouse KL DSE

Table S9 Gene ontology of mouse KL DSG

Table S10 MDS_Madan S34F alternative splicing

Table S11 MDS_Madan R156-Q157 alternative splicing

Table S12 MDS_Pellagatti S34F alternative splicing

Table S13 MDS_Pellagatti R156-Q157 alternative splicing

Table S14 AML_BeatAML S34F alternative splicing

Table S15 AML_BeatAML Q157 alternative splicing

Table S16 MDS-AML DSE

Table S17 Gene ontology of KL-MDS-AML shared DSG

Table S18 List of U2AF1 mutated patients identified in 21 studies

Table S19 Genomic data input for co-occurrence and mutual exclusivity analysis

Table S20 Co-occurrence and mutual exclusivity analysis from cBioPortal

Supplementary Methods and Figures

## Data Availability

Data are available on request to the corresponding author. RNA-seq data generated from this study have been deposited in the National Center for Biotechnology Information (NCBI) Gene Expression Omnibus (GEO) database (accession # GSE282060).
