# Supplementary material for: U2af1S34F and U2af1Q157R myeloid neoplasm-associated hotspot mutations induce distinct hematopoietic phenotypes in mice
Source: Leukemia. 2026 May 6;40(7):1467–81. doi: 10.1038/s41375-026-02974-7 (PMC13276503; doi:10.1038/s41375-026-02974-7)
Supplement: Supplementary file 21 — Supplementary Methods and Figures [file 41375_2026_2974_MOESM21_ESM.pdf]

SUPPLEMENTARY INFORMATION

U2af1^{S34F} and *U2af1*^{Q157R} myeloid neoplasm-associated hotspot mutations induce distinct hematopoietic phenotypes in mice.

Michael O. Alberti,[1,2] Sridhar Nonavinkere Srivatsan,[3] Jin Shao,[3] Dennis L. Fei,[4,5] Mengou Zhu,[3] Claudia Cabrera Pastrana,[3] Monique Chavez,[3] Stefan P. Tarnawsky,[3] Sarah Grieb,[3] Timothy A. Graubert,[6] Omar Abdel-Wahab,[7] and Matthew J. Walter[3]

[1]Department of Pathology and Immunology, Washington University, St. Louis, MO, USA; [2]Department of Pathology, University of Colorado Anschutz Medical Campus, Aurora, CO, USA; [3]Department of Medicine, Washington University, St. Louis, MO, USA; [4]Department of Medicine, Meyer Cancer Center, Weill Cornell Medicine, New York, NY, USA; [5]Cancer Biology Section, Cancer Genetics Branch, National Human Genome Research Institute, Bethesda, MD, USA; [6]Massachusetts General Hospital Cancer Center, Harvard Medical School, Boston, MA, USA; [7]Memorial Sloan Kettering Cancer Center, New York, NY, USA.

Table of Contents

Supplementary Methods 3

Supplementary References 7

List of Supplementary Tables (available on the *Leukemia* website) 10

Supplementary Fig. 1 11

Supplementary Fig. 2 14

Supplementary Fig. 3 16

Supplementary Fig. 4 18

Supplementary Fig. 5 19

Supplementary Fig. 6 21

Supplementary Fig. 7 23

Supplementary Fig. 8 25

Supplementary Methods

Generation *U2af1*$^{Q157R/+}$ (MG-Q157R) conditional knock-in mice

To create a germline conditional Q157R mutant allele at the mouse endogenous *U2af1* locus, we used a similar approach to that used for generating the MG-S34F mouse.[1] The MG-Q157R targeting vector was comprised of a wild-type (WT) MiniGene (encoding *U2af1* exons 4-8), three repeats of the SV40 late polyadenylation signal (3XpA), an FRT-flanked Neo cassette, and a mutated exon 6 carrying the Q157R missense mutation (CAG>CGG) (**Supplementary Fig. 1B**). LoxP sites flank the MG-3XpA-FRT-Neo-FRT cassette. The floxed MG-3XpA-FRT-Neo-FRT cassette was targeted to intron 3 (286 bp upstream of exon 4) using 5' (4.6 kb) and 3' (4.3 kb) homology arms to the *U2af1* locus. A diphtheria toxin A (DTA) cassette was included at the 3' end of the 3' homology arm to allow for negative selection. The targeting vector was constructed by Biocytogen (Waltham, MA) using standard PCR, Gibson, and recombineering (strain source: C57BL/6J) methods and verified by restriction enzyme digestion and Sanger sequencing. Linearized (via AscI restriction digest) targeting vector DNA was electroporated into B1-6 embryonic stem (ES) cells (Biocytogen). The B1-6 ES cell line is derived from C57BL/6J mice. ES cell clones surviving G418 selection were expanded for PCR screening followed by Southern blot (**Supplementary Fig. 1C**) and chromosome analyses to confirm correct homologous recombination and normal karyotype, respectively. Correctly targeted ES cell clones were microinjected into Balb/c blastocysts with resultant chimeric mice used to mate with C57BL/6J mice (Jackson Labs [JAX] Stock #000664, Bar Harbor, ME) to determine and establish germline transmission. F1 mice carrying the MG-Q157R allele (genotyping primers are listed in **Supplementary Table 1**) were crossed to *ACTB-FLPe* mice (JAX Stock #005703)[2] to remove the *Neo* cassette prior to subsequent breeding to existing C57BL/6 lines (e.g., *Mx1-Cre*).

Bone marrow (BM) transplant

CD45.1 male recipient mice were lethally irradiated (11 Gy delivered by Cs-source gamma-irradiator) the day before transplant of 2×10^6 whole BM CD45.2 donor cells by retroorbital injection. For competitive transplants, 1×10^6 CD45.1/CD45.2 whole BM 'competitor' cells were mixed with 1×10^6 CD45.2 whole BM 'test' cells prior to retroorbital injection. Recipient mice were maintained on TMP-SMX supplemented water for two weeks after transplant. Unless otherwise noted, six weeks post-transplant, mice were treated with three doses (300 µg each) of polyinosinic-polycytidylic acid (pIpC; GE Life Sciences/Cytiva, Marlborough, MA) by intraperitoneal (i.p.) injection every other day to induce *Mx1-Cre* expression.

Peripheral blood (PB) sampling and complete blood count (CBC)

PB was collected by capillary sampling of the retroorbital space to EDTA-coated collection tubes (Sarstedt, Nümbrecht, Germany). CBC for WBC (white blood cells), RBC (red blood cells), PLT (platelets), Hb (hemoglobin), and MCV (mean corpuscular volume) was performed on a Hemavet instrument (Drew Scientific, Plantation, FL).

Flow cytometry

Antibody staining for hematopoietic stem and progenitor cell (HSPC) and lineage or erythroid progenitor (EryP) panels was performed in FACS buffer (PBS supplemented with 2% fetal bovine serum and 10 mM EDTA) and analyzed on a ZT5/Yeti (Bio-Rad, Hercules, CA) and Attune (Thermo-Fisher, Waltham, MA) flow cytometers, respectively. Fluorescence-activated cell sorting (FACS) was performed on a highly modified MoFlo (Beckman Coulter, Brea, CA). Prior to sorting, Kit$^+$ cells were enriched from BM samples using CD117 (Kit) MicroBeads with the autoMACS Pro Separator (Miltenyi Biotec, Auburn, CA).

BM HSPC staining was performed with antibodies against the following: Gr-1, B220, CD3e, TER-119 (RB6-8C5, RA3-6B2, 145-2C11, TER-119 respectively; BV605; BioLegend, San Diego, CA), CD117/Kit (2B8; BV421; BioLegend), Sca-1 (D7; PE; BioLegend), CD48 (HM48-1; PE-Cy7; BioLegend), CD150 (TC15-12F12.2; APC-Fire750; BioLegend), CD34 (RAM34; FITC; eBioscience (Thermo-Fisher), Waltham, MA), CD16/32 (93; BV411; BioLegend), CD135 (A2F10; APC; eBioscience), CD45.1 (A20; BUV737; BD), CD45.2 (104; BUV395; BD). Lineage staining was performed with antibodies against the following: Gr-1 (RB6-8C5; APC-eFluor780;

eBioscience), CD11b (M1/70; BV421; BioLegend), CD115 (AFS98; PE; eBioscience), B220 (RA3-6B2; BV605; BioLegend), CD3e (145-2C11; APC; eBioscience), CD45.1 (A20; PE-Cy7 or eFluor450; BioLegend or eBioscience), CD45.2 (104; BV785 or FITC; BioLegend or eBioscience). EryP staining of spleen and BM (without erythrocyte lysis) was performed with antibodies against the following: Gr-1, B220, CD3e, (RB6-8C5, RA3-6B2, 145-2C11; BV605; BioLegend), TER-119 (TER-119; BV421; BioLegend), CD71 (YTA74.4; AF647; Bio-Rad), CD45.2 (104; BV785; BioLegend).

HSPC were broadly defined as KLS (Kit^{+}Lineage^{-}Sca-1^{+}) or KL (Kit^{+}Lineage^{-}Sca-1^{-}). KLS sub-populations were defined as long- and short-term hematopoietic stem cells (LT-HSC, CD135^{-}CD48^{-}CD150^{+}CD34^{-}; ST-HSC, CD135^{-}CD48^{-}CD150^{-}) and multipotent progenitors (MPP2, CD135^{-}CD48^{+}CD150^{+}; MPP3, CD135^{-}CD48^{+}CD150^{-}; and MPP4, CD135^{+}CD150^{-}).[3] KL sub-populations were defined as common myeloid progenitors (CMP, CD34^{+}CD16/32^{-}), granulocyte-macrophage progenitors (GMP, CD34^{+}CD16/32^{+}), and megakaryocyte-erythrocyte progenitors (MEP, CD34^{-}CD16/32^{-}).[4] Mature cell lineages were broadly defined as myeloid (CD11b^{+}) and lymphoid (CD11b^{-}B220^{+} and CD11b^{-}CD3^{+}), or as neutrophils (CD11b^{+}Gr-1^{+}SSC^{hi}), monocytes (CD11b^{+}CD115^{+}SSC^{lo}), B-cells (CD11b^{-}B220^{+}), and T-cells (CD11b^{-}CD3^{+}). See **Supplementary Fig. 1** for EryP gating strategy.[5]

Detection of *U2af1*^{S34F} and *U2af1*^{Q157R} mRNA expression using targeted NGS amplicon sequencing

BM KL cells were sorted into FACS buffer and gDNA-depleted total RNA were purified from cell pellets using the NucleoSpin RNA Plus XS Kit (Macherey-Nagel, Allentown, PA) in RNase-free water. RNA was treated with Ambion TURBO DNase (Thermo-Fisher) to remove genomic DNA contamination and then cDNA was prepared using the Invitrogen SuperScript III Reverse Transcriptase Kit (Thermo-Fisher) for subsequent two-step PCR. Primers with universal overhangs/tails were first used to separately amplify each of the regions centered on the S34 and Q157 codons of *U2af1*. Tailed amplicons were subsequently dual-indexed using i5 (6-mer) and i7 (8-mer) primers in a second PCR, multiplexed, and then gel purified (2% agarose/1X TAE) and diluted for 2x150 (S34) or 2x250 (Q157) sequencing on a MiSeq (Illumina, San Diego, CA) at the DNA Sequencing Innovation Lab at the Washington University Center for Genome Sciences & Systems Biology (CGS&SB). EconoTaq PLUS 2X Master Mix (LGC Biosearch Technologies, Middlesex, UK) was used for both PCR steps. For PCR step 1: 94°C for 2 min, followed by 20 cycles of 94°C for 15 sec, 55°C for 15 sec, and 72°C for 40 sec, and then 72°C for 2 min; 0.5 µM each primer. For PCR step 2: 94°C for 2 min, followed by 10 cycles of 94°C for 30 sec, 55°C for 30 sec, and 72°C for 40 sec, and then 72°C for 2 min; 0.5 µM i5 and 1.25 µM i7 primers. All primer sequences are listed in **Supplementary Table 1**. The ratios of WT to S34F reads and WT to Q157R and Q157Rdel reads were determined using the grep command on demultiplexed 2x150 or 2x250 FASTQ files, respectively.

2x150 FASTQ file grep search strings (S34 codon underlined):

u2af1_wt	TGTCGTCATGGAGACAGATGTTCTCGGTTGCACAATAAACCAACC
u2af1_s34f	TGTCGTCATGGAGACAGATGTTTTCGGTTGCACAATAAACCAACC

2x250 FASTQ file grep search strings (Q157 codon underlined):

u2af1_wt	TGTGCACTCTCCCATTTCATACTGGCGGCAGCAGGCTTCCCTGAA
u2af1_q157r	TGTGCACTCTCCCATTTCATACCGGCGGCAGCAGGCTTCCCTGAA
u2af1_q157rdel	TGTGCACTCTCGGCGGCAGCAGGCTTCCCTGAAGTCAGTTAC

RNA-seq bioinformatics for mouse KL cell samples and code availability

After mouse KL cDNA library amplification, approximately 360 bp fragments were sequenced on an Illumina NovaSeq 6000 instrument. Reads were mapped to the mouse genome (GRCm38 version from Ensembl) using STAR (v2.7.0; ref. 6). Following alignment, TPM values for internal QC of *Kit* and *Ly6a* expression were calculated for all samples using StringTie (v1.3.3; ref. 7). In parallel, gene level counts for mutant and control samples were processed using kallisto (v0.43.1; ref. 8). Genes with ≥5 reads in at least half of the samples were retained for subsequent analysis. Normalized gene values (CPM) were used for unsupervised principal component analysis (PCA). Differentially expressed genes (DEG) were identified using DESeq2 (v1.26.0; ref. 9) with $|\log_2 FC|>1$ and FDR<0.05 as thresholds for significance. Gene ontology (GO) analysis of either upregulated or downregulated DEG was performed using clusterProfiler (v3.10.1; ref. 10) with FDR (q-value)<0.1 as a threshold for significance. REVIGO (v1.8.1; ref. 11) was used to consolidate gene sets into a representative

subset of GO terms. Pathway enrichment analysis was performed using fgsea (v1.17.0; https://doi.org/doi:10.18129/B9.bioc.fgsea) against GO, Reactome, and MSigDB gene sets with FDR<0.1 as a threshold for significance.

Following alignment, rMATS-turbo (v4.1.1; ref. 12) was also used to detect annotated alternative 3' or 5' splice site (A3SS, A5SS), mutually exclusive exon (MXE), retained intron (RI), and skipped exon (SE) events and significant splicing differences (based on the difference of percent spliced-in [ΔPSI] values) between mutant and control samples. Positive (also referred to as '↑') and negative (also referred to as '↓') ΔPSI values indicate inclusion and exclusion, respectively, of a splicing event relative to control. Events with ≥5 reads supporting each isoform in at least half of the samples were retained for subsequent analysis including for unsupervised PCA. Differentially spliced events (DSE) were identified with |ΔPSI|>0.05 and FDR<0.05 as thresholds for significance. GO analysis of DSE was performed using clusterProfiler and REVIGO, as above. Logos plots were created using SeqLogo (v.1.70.0; https://doi.org/doi:10.18129/B9.bioc.seqLogo). The sashimi plot of the A5SS event in exon 6 of *U2af1* (created by the Q157R mutation) was generated using rmats2sashimiplots (v3.0).

The variant allele frequencies (VAF) of the *U2af1* S34F (C>T) and Q157R (A>G) mutations in mouse RNA-seq samples were calculated by counting reference and alternate alleles at the corresponding nucleotide positions (chr17:31655010 and chr17:31648195) using bam-readcount.[13]

The mouse BM myeloid progenitor cell dataset from Fei *et al.*[1] (GSE112174) was reanalyzed using the above methods/pipelines.

RNA-seq data generated from this study have been deposited in the National Center for Biotechnology Information (NCBI) Gene Expression Omnibus (GEO) database (accession # GSE282060).

Bioinformatics and reanalysis of published MDS and AML RNA-seq datasets

MDS and AML datasets from Madan *et al.*[14] (GSE128429), Pellagatti *et al.*[15] (GSE114922), and Beat AML[16] (phs001657.v1.p1) were reanalyzed using the above methods, except reads were mapped to the human genome (Homo_sapiens.GRCh38.95.gtf)[17] using STAR (v2.7.0) prior to analysis of alternative splicing using rMATS-turbo (v.4.1.1). In each of the three studies, samples from MDS/AML patients who were reported to not have identifiable mutations in *SF3B1* or *SRSF2* were used as a comparator (Splicing Factor [SF]^{WT}). The variant allele frequencies (VAF) of the *U2AF1* S34F (C>T), R156H (G>A), Q157P (A>C), and Q157R (A>G) mutations in MDS/AML RNA-seq samples from all three studies were calculated by counting reference and alternate alleles at the corresponding nucleotide positions (chr21:43104346, chr21:43094670, and chr21:43094667) using bam-readcount, as above.

Confirmation of splicing changes

For confirmation of splicing changes at 4 weeks post-pIpC, BM KL cells were sorted into FACS buffer and gDNA-depleted total RNA were purified from cell pellets using the NucleoSpin RNA Plus XS Kit (Macherey-Nagel) in RNase-free water. For confirmation of splicing changes at 24 weeks post-pIpC, Kit+ cells were enriched from BM samples using the autoMACS Pro Separator (as above) and total RNA was isolated using TRIzol reagent (Invitrogen (Thermo-Fisher)). Total RNA was isolated from MDS and s-AML patient cells using TRIzol reagent (Invitrogen (Thermo-Fisher)) by the Tissue Procurement Core at Washington University. RNA concentration was measured by Qubit Fluorometer (Thermo-Fisher). RNA was treated with Ambion TURBO DNase (Thermo-Fisher) to remove genomic DNA contamination and then cDNA was prepared using the Invitrogen SuperScript III Reverse Transcriptase Kit (Thermo-Fisher) for subsequent standard PCR (RT-PCR). Aberrant alternative splicing events were confirmed by PCR with EconoTaq PLUS Master Mix (LGC Biosearch Technologies) using cDNA input and standard cycling parameters (94°C for 3 min, followed by 38 cycles of 95°C for 30 sec, 60°C for 60 sec, and 72°C for 60 sec, and then 72°C for 5 min). PCR products were separated by polyacrylamide gel electrophoresis (1X TBE) and stained with ethidium bromide prior to imaging. Densitometry was performed using ImageJ. All primer sequences are listed in **Supplementary Table 1**. All patients provided written consent on a protocol approved by the WUSTL Human Studies Committee. Clinical characteristics of MDS and s-AML patients who donated research samples are listed in **Supplementary Table 2**.

Analysis of *U2AF1* hotspot mutation frequencies in myeloid malignancies

Patients with *U2AF1* mutations were identified in 21 published studies.[16,18–37] Patients with a *U2AF1* mutation(s) (i.e., S34[F/Y], R156H/Q157[P/R], both S34 and R156/Q157, or 'other' rare variants) and a diagnosis of AML (N= 50 patients), sAML (from MDS; N=51 patients), MDS (N=271 patients), CMML (N=47 patients), and MPN (N=68 patients), were included (**Supplementary Table 18**). Patients without a diagnosis or having an alternative diagnosis were excluded from the analysis.

Analysis of *U2AF1* hotspot mutation co-occurrence and mutual exclusivity in myeloid malignancies

Mutation data were included from 20 published studies that performed *U2AF1* sequencing and had patient-level mutation data available for a common set of genes sequenced across all studies for the specific disease category.[16,18–36] Mutations across all patients were pooled by disease group. For the TCGA AML cohort, a total of 20 patients had an *SRSF2* mutation, one patient reported in the original publication[22] and an additional 19 patients identified using RNA-seq data.[38] Patients with a diagnosis of AML (N=1857 patients), sAML (from MDS; N=458 patients), MDS (N=3159 patients), CMML (N=430 patients), and MPN (N=1551 patients) were included regardless of their mutation status. Patients who did not have a detected mutation in the targeted gene panel and did not have a unique patient identifier reported were also included in the analysis (sAML [N=24 patients], MDS [N=107 patients], and MPN [N=82 patients]) (see **Supplemental Table 19** for study details). Genes included in the mutation co-occurrence and mutual-exclusivity analysis were sequenced across all studies within each disease group. Coding and splice region variants in thirty-one genes for AML, sAML, MDS, and CMML groups and 23 genes for the MPN group were included in the analysis (**Supplementary Table 19**). cBioPortal (www.cbioportal.org) was used for the co-occurrence and mutual-exclusivity of genomic alteration analysis within each disease group using the default settings.[39–41] cBioPortal output data are available in **Supplementary Table 20** and represented in **Fig. 8B** and **Supplementary Fig. 8**.

Supplementary References

1. Fei DL, Zhen T, Durham B, et al. Impaired hematopoiesis and leukemia development in mice with a conditional knock-in allele of a mutant splicing factor gene U2af1. *Proc Natl Acad Sci U S A*. 2018;115(44):E10437-E10446. doi:10.1073/pnas.1812669115

2. Rodríguez CI, Buchholz F, Galloway J, et al. High-efficiency deleter mice show that FLPe is an alternative to Cre-loxP. *Nat Genet*. 2000;25(2):139-140. doi:10.1038/75973

3. Pietras EM, Reynaud D, Kang YA, et al. Functionally Distinct Subsets of Lineage-Biased Multipotent Progenitors Control Blood Production in Normal and Regenerative Conditions. *Cell Stem Cell*. 2015;17(1):35-46. doi:10.1016/j.stem.2015.05.003

4. Akashi K, Traver D, Miyamoto T, Weissman IL. A clonogenic common myeloid progenitor that gives rise to all myeloid lineages. *Nature*. 2000;404(6774):193-197. doi:10.1038/35004599

5. Socolovsky M, Nam H, Fleming MD, Haase VH, Brugnara C, Lodish HF. Ineffective erythropoiesis in Stat5a(-/-)5b(-/-) mice due to decreased survival of early erythroblasts. *Blood*. 2001;98(12):3261-3273. doi:10.1182/blood.v98.12.3261

6. Dobin A, Davis CA, Schlesinger F, et al. STAR: ultrafast universal RNA-seq aligner. *Bioinforma Oxf Engl*. 2013;29(1):15-21. doi:10.1093/bioinformatics/bts635

7. Pertea M, Pertea GM, Antonescu CM, Chang TC, Mendell JT, Salzberg SL. StringTie enables improved reconstruction of a transcriptome from RNA-seq reads. *Nat Biotechnol*. 2015;33(3):290-295. doi:10.1038/nbt.3122

8. Bray NL, Pimentel H, Melsted P, Pachter L. Near-optimal probabilistic RNA-seq quantification. *Nat Biotechnol*. 2016;34(5):525-527. doi:10.1038/nbt.3519

9. Love MI, Huber W, Anders S. Moderated estimation of fold change and dispersion for RNA-seq data with DESeq2. *Genome Biol*. 2014;15(12):550. doi:10.1186/s13059-014-0550-8

10. Yu G, Wang LG, Han Y, He QY. clusterProfiler: an R package for comparing biological themes among gene clusters. *Omics J Integr Biol*. 2012;16(5):284-287. doi:10.1089/omi.2011.0118

11. Supek F, Bošnjak M, Škunca N, Šmuc T. REVIGO summarizes and visualizes long lists of gene ontology terms. *PloS One*. 2011;6(7):e21800. doi:10.1371/journal.pone.0021800

12. Shen S, Park JW, Lu Z xiang, et al. rMATS: robust and flexible detection of differential alternative splicing from replicate RNA-Seq data. *Proc Natl Acad Sci U S A*. 2014;111(51):E5593-5601. doi:10.1073/pnas.1419161111

13. Khanna A, Larson DE, Srivatsan SN, et al. Bam-readcount - rapid generation of basepair-resolution sequence metrics. *ArXiv*. Published online 27 July 2021:arXiv:2107.12817v1.

14. Madan V, Li J, Zhou S, et al. Distinct and convergent consequences of splice factor mutations in myelodysplastic syndromes. *Am J Hematol*. 2020;95(2):133-143. doi:10.1002/ajh.25673

15. Pellagatti A, Armstrong RN, Steeples V, et al. Impact of spliceosome mutations on RNA splicing in myelodysplasia: dysregulated genes/pathways and clinical associations. *Blood*. 2018;132(12):1225-1240. doi:10.1182/blood-2018-04-843771

16. Tyner JW, Tognon CE, Bottomly D, et al. Functional genomic landscape of acute myeloid leukaemia. *Nature*. 2018;562(7728):526-531. doi:10.1038/s41586-018-0623-z

17. Miller CA, Walker JR, Jensen TL, et al. Failure to Detect Mutations in U2AF1 due to Changes in the GRCh38 Reference Sequence. *J Mol Diagn JMD*. 2022;24(3):219-223. doi:10.1016/j.jmoldx.2021.10.013

18. Papaemmanuil E, Gerstung M, Malcovati L, et al. Clinical and biological implications of driver mutations in myelodysplastic syndromes. *Blood*. 2013;122(22):3616-3627; quiz 3699. doi:10.1182/blood-2013-08-518886

19. Lindsley RC, Mar BG, Mazzola E, et al. Acute myeloid leukemia ontogeny is defined by distinct somatic mutations. *Blood*. 2015;125(9):1367-1376. doi:10.1182/blood-2014-11-610543

20. Yoshida K, Sanada M, Shiraishi Y, et al. Frequent pathway mutations of splicing machinery in myelodysplasia. *Nature*. 2011;478(7367):64-69. doi:10.1038/nature10496

21. Walter MJ, Shen D, Shao J, et al. Clonal diversity of recurrently mutated genes in myelodysplastic syndromes. *Leukemia*. 2013;27(6):1275-1282. doi:10.1038/leu.2013.58

22. Cancer Genome Atlas Research Network, Ley TJ, Miller C, et al. Genomic and epigenomic landscapes of adult de novo acute myeloid leukemia. *N Engl J Med*. 2013;368(22):2059-2074. doi:10.1056/NEJMoa1301689

23. Bernard E, Tuechler H, Greenberg PL, et al. Molecular International Prognostic Scoring System for Myelodysplastic Syndromes. *NEJM Evid*. 2022;1(7):EVIDoa2200008. doi:10.1056/EVIDoa2200008

24. Welch JS, Petti AA, Miller CA, et al. TP53 and Decitabine in Acute Myeloid Leukemia and Myelodysplastic Syndromes. *N Engl J Med*. 2016;375(21):2023-2036. doi:10.1056/NEJMoa1605949

25. Tefferi A, Lasho TL, Finke CM, et al. Targeted deep sequencing in primary myelofibrosis. *Blood Adv*. 2016;1(2):105-111. doi:10.1182/bloodadvances.2016000208

26. Papaemmanuil E, Gerstung M, Bullinger L, et al. Genomic Classification and Prognosis in Acute Myeloid Leukemia. *N Engl J Med*. 2016;374(23):2209-2221. doi:10.1056/NEJMoa1516192

27. Kim T, Tyndel MS, Kim HJ, et al. The clonal origins of leukemic progression of myelodysplasia. *Leukemia*. 2017;31(9):1928-1935. doi:10.1038/leu.2017.17

28. Merlevede J, Droin N, Qin T, et al. Mutation allele burden remains unchanged in chronic myelomonocytic leukaemia responding to hypomethylating agents. *Nat Commun*. 2016;7:10767. doi:10.1038/ncomms10767

29. Menssen AJ, Khanna A, Miller CA, et al. Convergent Clonal Evolution of Signaling Gene Mutations Is a Hallmark of Myelodysplastic Syndrome Progression. *Blood Cancer Discov*. 2022;3(4):330-345. doi:10.1158/2643-3230.BCD-21-0155

30. Guess T, Potts CR, Bhat P, et al. Distinct Patterns of Clonal Evolution Drive Myelodysplastic Syndrome Progression to Secondary Acute Myeloid Leukemia. *Blood Cancer Discov*. 2022;3(4):316-329. doi:10.1158/2643-3230.BCD-21-0128

31. Duncavage EJ, Jacoby MA, Chang GS, et al. Mutation Clearance after Transplantation for Myelodysplastic Syndrome. *N Engl J Med*. 2018;379(11):1028-1041. doi:10.1056/NEJMoa1804714

32. McNamara CJ, Panzarella T, Kennedy JA, et al. The mutational landscape of accelerated- and blast-phase myeloproliferative neoplasms impacts patient outcomes. *Blood Adv*. 2018;2(20):2658-2671. doi:10.1182/bloodadvances.2018021469

33. Zhang H, Wilmot B, Bottomly D, et al. Genomic landscape of neutrophilic leukemias of ambiguous diagnosis. *Blood*. 2019;134(11):867-879. doi:10.1182/blood.2019000611

34. Tamari R, Rapaport F, Zhang N, et al. Impact of High-Molecular-Risk Mutations on Transplantation Outcomes in Patients with Myelofibrosis. *Biol Blood Marrow Transplant J Am Soc Blood Marrow Transplant*. 2019;25(6):1142-1151. doi:10.1016/j.bbmt.2019.01.002

35. Tefferi A, Guglielmelli P, Lasho TL, et al. Mutation-enhanced international prognostic systems for essential thrombocythaemia and polycythaemia vera. *Br J Haematol*. 2020;189(2):291-302. doi:10.1111/bjh.16380

36. Nangalia J, Massie CE, Baxter EJ, et al. Somatic CALR mutations in myeloproliferative neoplasms with nonmutated JAK2. *N Engl J Med*. 2013;369(25):2391-2405. doi:10.1056/NEJMoa1312542

37. Patnaik MM, Lasho TL, Finke CM, et al. Spliceosome mutations involving SRSF2, SF3B1, and U2AF35 in chronic myelomonocytic leukemia: prevalence, clinical correlates, and prognostic relevance. *Am J Hematol*. 2013;88(3):201-206. doi:10.1002/ajh.23373

38. Yoshimi A, Lin KT, Wiseman DH, et al. Coordinated alterations in RNA splicing and epigenetic regulation drive leukaemogenesis. *Nature*. 2019;574(7777):273-277. doi:10.1038/s41586-019-1618-0

39. Cerami E, Gao J, Dogrusoz U, et al. The cBio cancer genomics portal: an open platform for exploring multidimensional cancer genomics data. *Cancer Discov*. 2012;2(5):401-404. doi:10.1158/2159-8290.CD-12-0095

40. Gao J, Aksoy BA, Dogrusoz U, et al. Integrative analysis of complex cancer genomics and clinical profiles using the cBioPortal. *Sci Signal*. 2013;6(269):pl1. doi:10.1126/scisignal.2004088

41. de Bruijn I, Kundra R, Mastrogiacomo B, et al. Analysis and Visualization of Longitudinal Genomic and Clinical Data from the AACR Project GENIE Biopharma Collaborative in cBioPortal. *Cancer Res*. 2023;83(23):3861-3867. doi:10.1158/0008-5472.CAN-23-0816

List of Supplementary Tables

Supplementary Table 1. List of primers.

Supplementary Table 2. Clinical characteristics of patients who donated MDS and sAML samples for this study.

Supplementary Table 3. Unfiltered gene expression analysis for *U2af1*$^{S34F/+}$ and *U2af1*$^{Q157R/+}$ KL cells.

Supplementary Table 4. Gene ontology analysis of DEG for *U2af1*$^{S34F/+}$ and *U2af1*$^{Q157R/+}$ KL cells.

Supplementary Table 5. Unfiltered rMATS analysis for *U2af1*$^{S34F/+}$ vs *U2af1*$^{+/+}$ KL cells.

Supplementary Table 6. Unfiltered rMATS analysis for *U2af1*$^{Q157R/+}$ vs *U2af1*$^{+/+}$ KL cells.

Supplementary Table 7. Unfiltered rMATS analysis for *U2af1*$^{S34F/+}$ vs *U2af1*$^{+/+}$ KL (Fei) cells.

Supplementary Table 8. DSE identified in *U2af1*$^{S34F/+}$ and *U2af1*$^{Q157R/+}$ KL cells.

Supplementary Table 9. Gene ontology analysis of DSG for *U2af1*$^{S34F/+}$ and *U2af1*$^{Q157R/+}$ KL cells.

Supplementary Table 10. Unfiltered rMATS analysis for *U2AF1*^{S34F} vs SF^{WT} MDS cells (Madan).

Supplementary Table 11. Unfiltered rMATS analysis for *U2AF1*$^{R156/Q157}$ vs SF^{WT} MDS cells (Madan).

Supplementary Table 12. Unfiltered rMATS analysis for *U2AF1*^{S34F} vs SF^{WT} MDS cells (Pellagatti).

Supplementary Table 13. Unfiltered rMATS analysis for *U2AF1*$^{R156/Q157}$ vs SF^{WT} MDS cells (Pellagatti).

Supplementary Table 14. Unfiltered rMATS analysis for *U2AF1*^{S34F} vs SF^{WT} AML cells (Beat AML).

Supplementary Table 15. Unfiltered rMATS analysis for *U2AF1*^{Q157} vs SF^{WT} AML cells (Beat AML).

Supplementary Table 16. DSE identified in *U2AF1*^{S34F} and *U2AF1*$^{R156/Q157}$ MDS and AML cells.

Supplementary Table 17. Gene ontology analysis of shared MDS/AML and KL DSG for S34F and R156/Q157.

Supplementary Table 18. List of patients with *U2AF1* mutations from 21 studies.

Supplementary Table 19. Genomic data input for co-occurrence and mutual exclusivity analysis.

Supplementary Table 20. Unfiltered co-occurrence and mutual exclusivity analysis from cBioPortal.

Supplementary Fig. 1

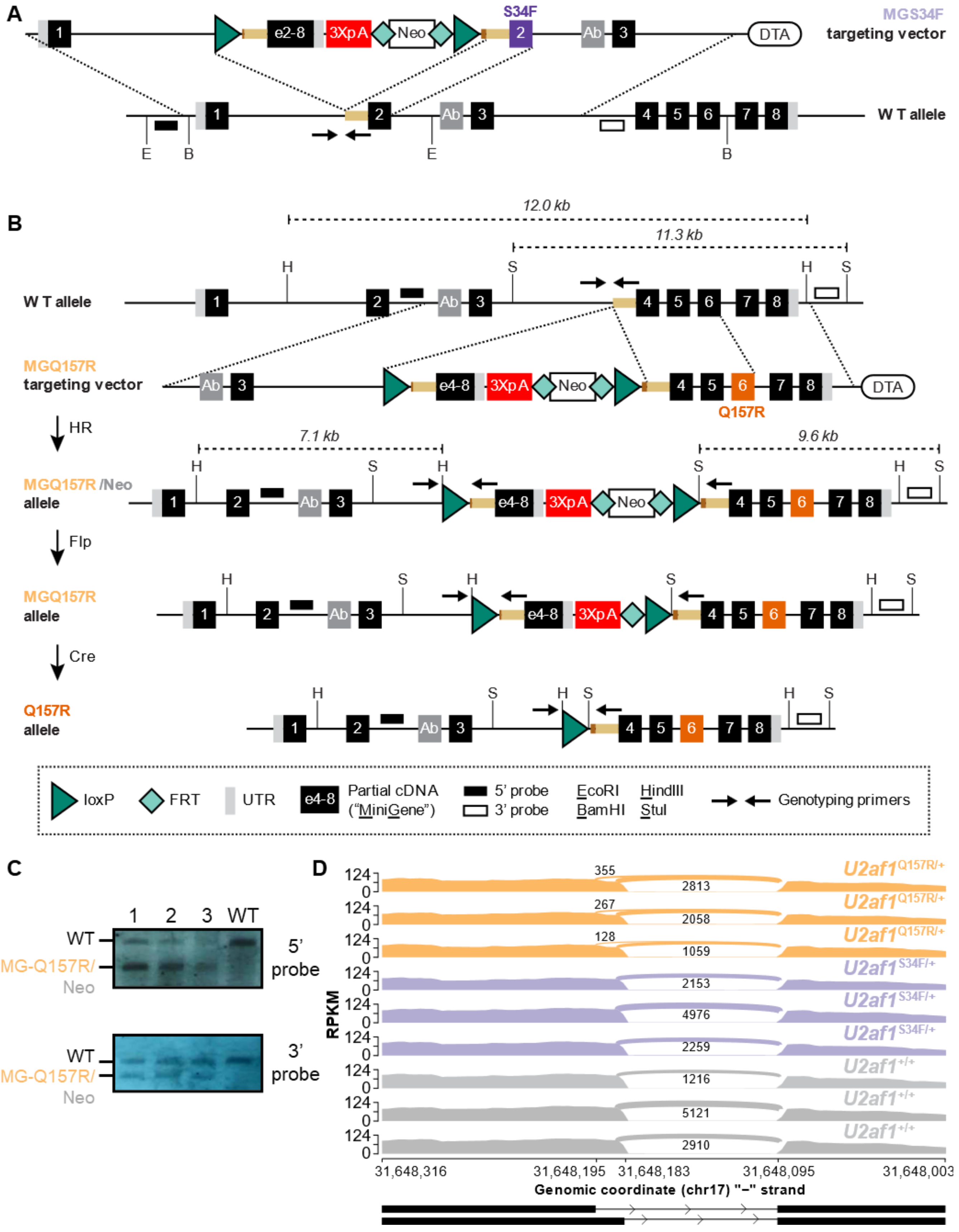

Supplementary Fig. 1

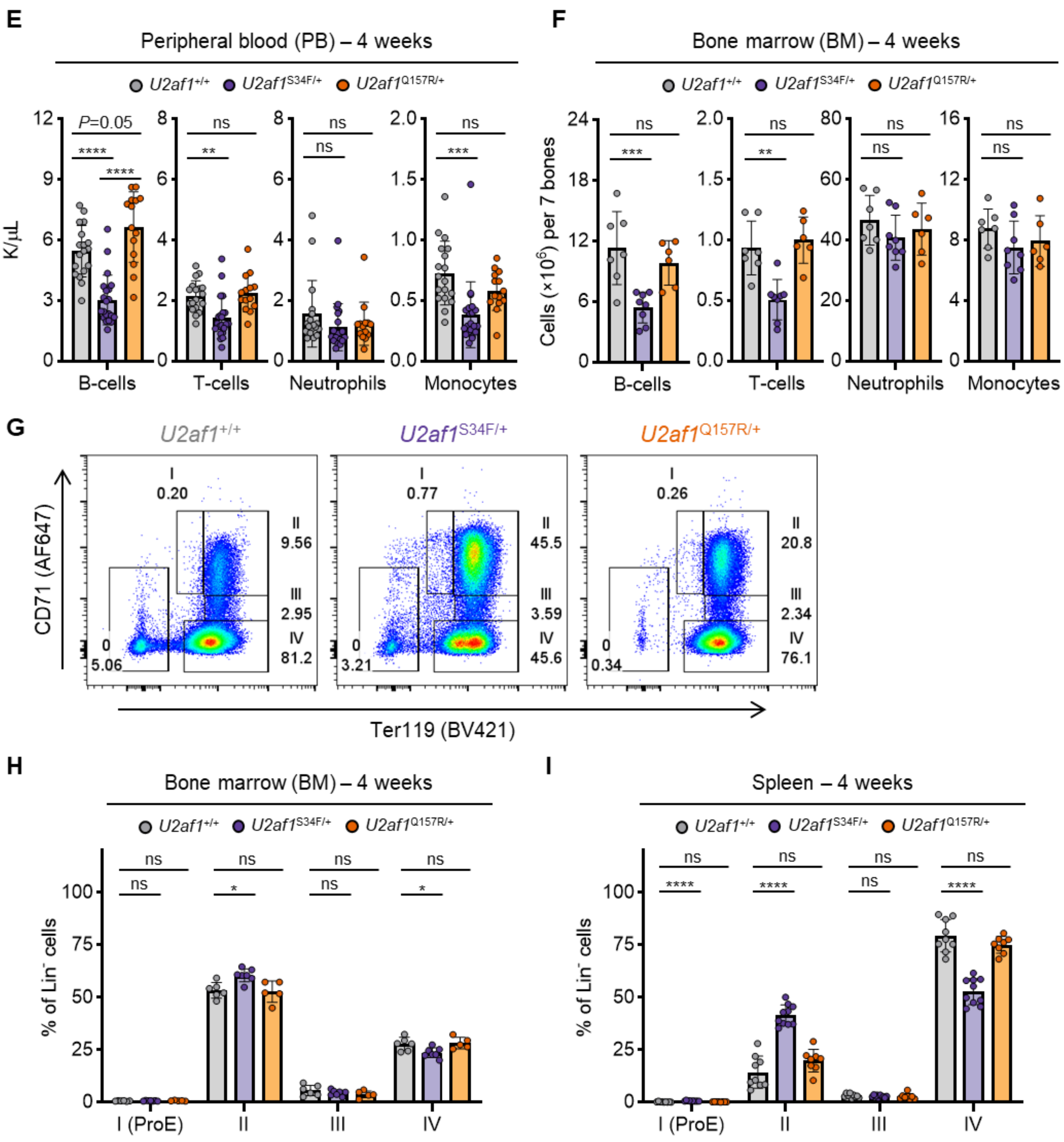

Supplementary Fig. 1. Related to Fig. 1. (**A-D**) Derivation of *U2af1*$^{S34F/+}$ and *U2af1*$^{Q157R/+}$ conditional knock-in mice. (**A**) Diagram of the MGS34F targeting vector used to create a conditional S34F mutant allele at the mouse endogenous *U2af1* locus (WT allele) previously reported in Fei *et al.*[1] The MG-S34F targeting vector contains a WT MiniGene (MG; encoding *U2af1* exons 2-8), three repeats of the SV40 late polyadenylation signal (3XpA), an FRT-flanked Neo cassette, and a mutated exon 2 carrying the S34F missense mutation (TCT>TTT). LoxP sites flank the MG-3XpA-FRT-Neo-FRT cassette. The targeted allele (after removal of Neo by FLPe) is shown in **Fig. 1B**. Diagram adapted from Fei *et al.*[1] (**B**) Diagram of the MGQ157R targeting vector used to create a conditional Q157R mutant allele at the mouse endogenous *U2af1* locus (WT allele) in this study. The strategy is similar to that used to make the MGS34F mouse in (**A**). The MGQ157R targeting vector contains a WT MG (encoding *U2af1* exons 4-8), three repeats of the SV40 late polyadenylation signal (3XpA), an FRT-flanked Neo cassette, and a mutated exon 6 carrying the Q157R missense mutation (CAG>CGG). LoxP sites flank the MG-3XpA-FRT-Neo-FRT cassette. After successful targeting of the MGQ157R/Neo allele to the *U2af1* locus to generate F1 mice, the Neo cassette was removed by crossing to FLPe-expressing mice. Restriction sites used for Southern blotting and primer binding sites used for PCR genotyping are shown. Cre-mediated recombination of the MGQ157R allele results in removal of the WT MG cassette and conditional expression of *U2af1*^{Q157R} from the mouse endogenous locus. See also **Fig. 1C**. (**C**) Southern blot using genomic DNA from ES cells to demonstrate successful targeting of the MGQ157R/Neo allele to the endogenous *U2af1* locus. Genomic DNA was prepared for Southern blot by cutting with either HindIII (for 5'-probe; top) or StuI (for 3'-probe; bottom). Results for three positive ES cell clones and the parental WT ES cell line are shown. (**D**) Sashimi plot of the alternative 5' splice site event in exon 6 of *U2af1* that is created by the Q157R mutation. (**E-I**) Additional characterization of native hematopoiesis in *U2af1*$^{S34F/+}$ and *U2af1*$^{Q157R/+}$ conditional knock-in mice. (**E**) Flow cytometric analysis of PB samples at 4 weeks post-pIpC to determine absolute counts of lymphoid (B-cells or T-cells) and myeloid (Neutrophils or Monocytes) cells. N=14-21 mice per genotype pooled from four independent experiments. (**F**) Flow cytometric analysis of BM samples at 4 weeks post-pIpC to determine absolute counts of lymphoid (B-cells or T-cells) and myeloid (Neutrophils or Monocytes) cells. N=6-8 mice per genotype pooled from two independent experiments. (**G-I**) Representative flow plots with gating schema (**G**) used to determine the relative distribution of erythroid progenitor (EryP) subsets (labeled I-IV)[5] in bone marrow (**H**) and spleen (**I**) determined by flow cytometric immunophenotyping at 4 weeks post-pIpC. N=5-7 (**H**) and 8-10 (**I**) mice per genotype pooled from two and three independent experiments, respectively. Results represent the mean ± standard deviation (SD) (**E-F**, and **H-I**). A one-way ANOVA with Tukey multiple comparison correction was used for the comparison of groups (**E-F**, and **H-I**). **P* < 0.05; ***P* < 0.01; ****P* < 0.001; *****P* < 0.0001. ns, not significant (or labeled if *P* < 0.10).

Supplementary Fig. 2

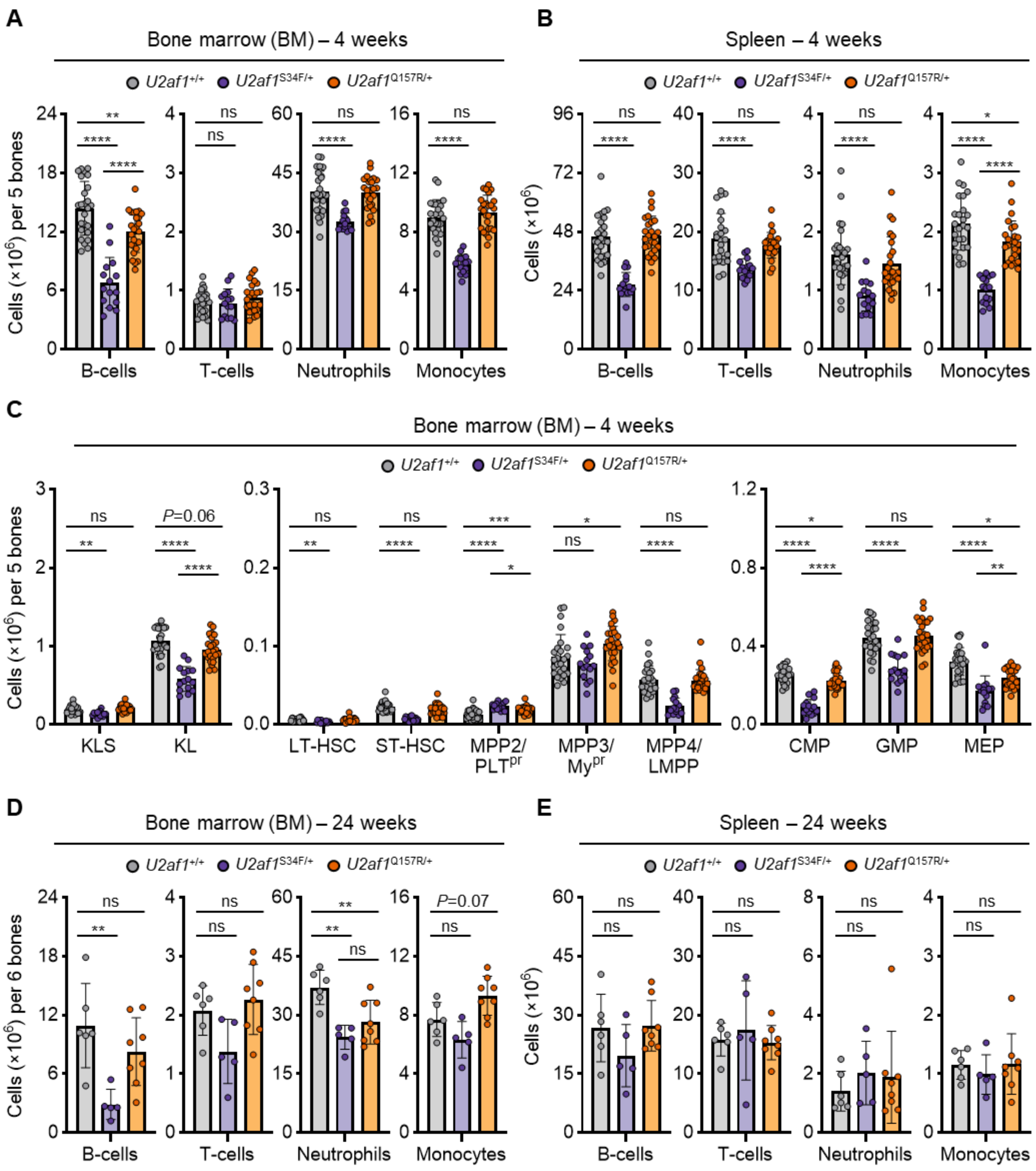

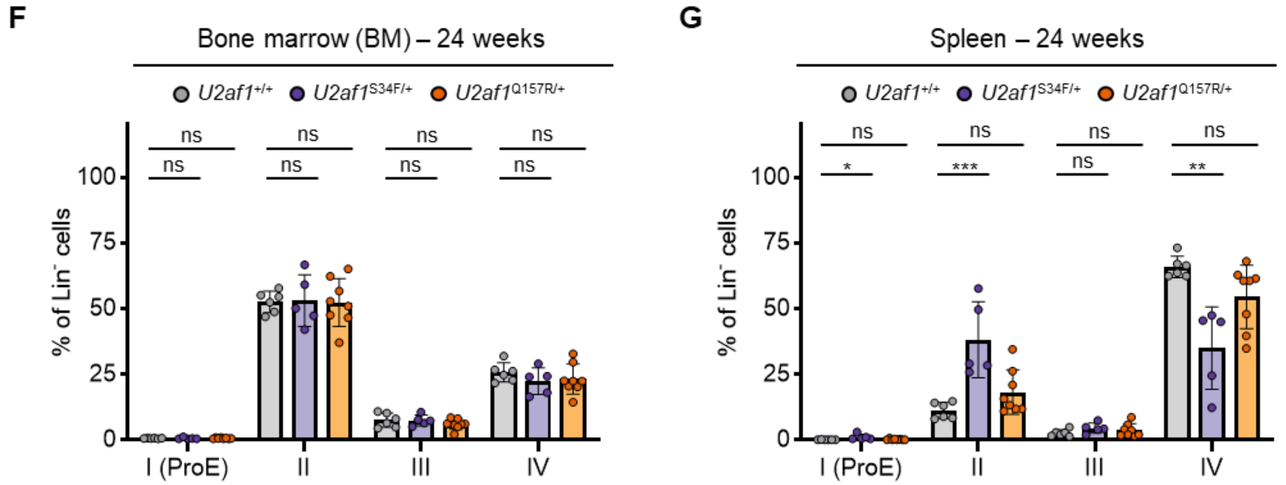

Supplementary Fig. 2. Related to Fig. 2. (**A-C**) As in **Fig. 2A**, CD45.2⁺ donor BM cells from *U2af1*$^{+/+}$, *U2af1*$^{S34F/+}$, or *U2af1*$^{Q157R/+}$ mice (all *Mx1-Cre*$^{+}$) were transplanted into lethally irradiated WT congenic (CD45.1^{+}) recipient mice. Recipient mice were treated with pIpC at 6 weeks post-transplant and BM and spleen were collected for flow cytometric analyses at 4 weeks post-pIpC. N=15-25 recipient mice from five independent experiments. (**A-B**) Flow cytometric analysis of BM (**A**) and spleen (**B**) was performed to determine absolute counts of lymphoid (B-cells or T-cells) and myeloid (neutrophils or monocytes) cells. (**C**) Absolute cell counts of BM HSPC populations (KLS [Kit^{+}Lineage^{-}Sca-1^{+}], KL [Kit^{+}Lineage^{-}Sca-1^{-}], long-term and short-term HSC [LT-HSC and ST-HSC], multipotent progenitors [MPP2, MPP3, and MPP4], common myeloid progenitors [CMP], granulocyte-macrophage progenitors [GMP], and megakaryocyte-erythrocyte progenitors [MEP]) in recipient mice were determined by flow cytometric analysis. (**D-G**) Related to **Fig. 2A-D**. Flow cytometric analysis of BM (**D**) and spleen (**E**) was performed to determine absolute counts of lymphoid and myeloid cells at 24 weeks post-pIpC. Relative distribution of erythroid progenitor (EryP) subsets (labeled I-IV as in **Supplementary Fig. 1H,I**) in BM (**F**) and spleen (**G**) determined by flow cytometric immunophenotyping at 24 weeks post-pIpC. N=5-8 recipient mice pooled from two independent experiments. Results represent the mean ± SD (**A-G**). A one-way ANOVA with Tukey multiple comparison correction was used for the comparison of groups (**A-G**). **P* < 0.05; ***P* < 0.01; ****P* < 0.001; *****P* < 0.0001. ns, not significant (or labeled if *P* < 0.10).

Supplementary Fig. 3

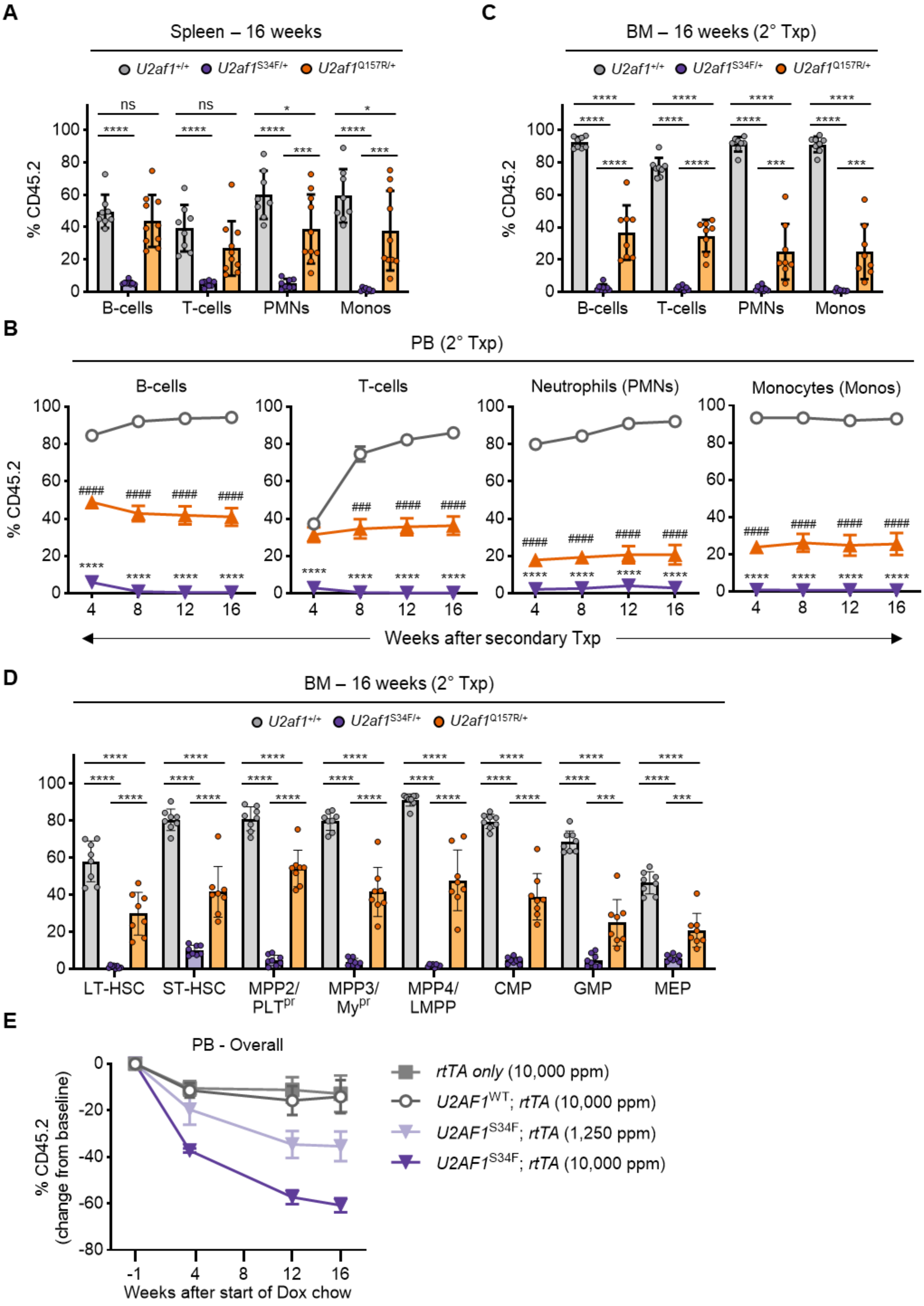

Supplementary Fig. 3. Related to Fig. 3. (**A**) Donor cell chimerism ($CD45.2^+$) of lymphoid (B-cells or T-cells) and myeloid (PMNs/neutrophils or monocytes) cell populations was assessed on spleen from recipient mice at 16 weeks post-pIpC. N=8-10 recipient mice pooled from two independent experiments. (**B**-**D**) Donor cell chimerism ($CD45.2^+$) was assessed on PB and BM from recipient mice receiving a secondary transplant. (**B**-**C**) Chimerism of lymphoid (B-cells or T-cells) and myeloid (Neutrophils or Monocytes) cell populations in the PB (**B**) and BM (**C**). (**D**) Chimerism of HSPC populations in BM. N=8 recipient mice per genotype from one experiment. (**E**) Competitive transplant assay using doxycycline (Dox)-inducible targeted (*Col1a1* locus) transgenic $U2AF1^{WT}$ and $U2AF1^{S34F}$ mice. $CD45.2^+$ (test) donor BM cells from *rtTA* only littermate controls, $U2AF1^{WT}$;*rtTA*, or $U2AF1^{S34F}$;*rtTA* mice were each mixed 1:1 with $CD45.1^+$/$CD45.2^+$ competitor BM cells and transplanted into lethally irradiated WT congenic ($CD45.1^+$) recipient mice. Recipient mice were fed Dox chow (1,250 ppm or 10,000 ppm) at 9 weeks post-transplant and donor cell chimerism ($CD45.2^+$) was assessed on PB from recipient mice before (−1 week) and up to 16 weeks post-Dox. Results represent the mean ± SEM (**B**,**E**) or mean ± SD (**A**,**C**, and **D**). A two-way ANOVA with repeated measures and Tukey multiple comparison correction (**B**) or one-way ANOVA with Tukey multiple comparison correction (**A**,**C**, and **D**) were used for the comparison of groups. *$P < 0.05$; ***$P < 0.001$; ****$P < 0.0001$. ns, not significant (or labeled if $P < 0.10$). Symbols ($U2af1^{+/+}$ vs $U2af1^{S34F/+}$ [*]; $U2af1^{+/+}$ vs $U2af1^{Q157R/+}$ [#]) are used to differentiate comparisons in **B**.

Supplementary Fig. 4

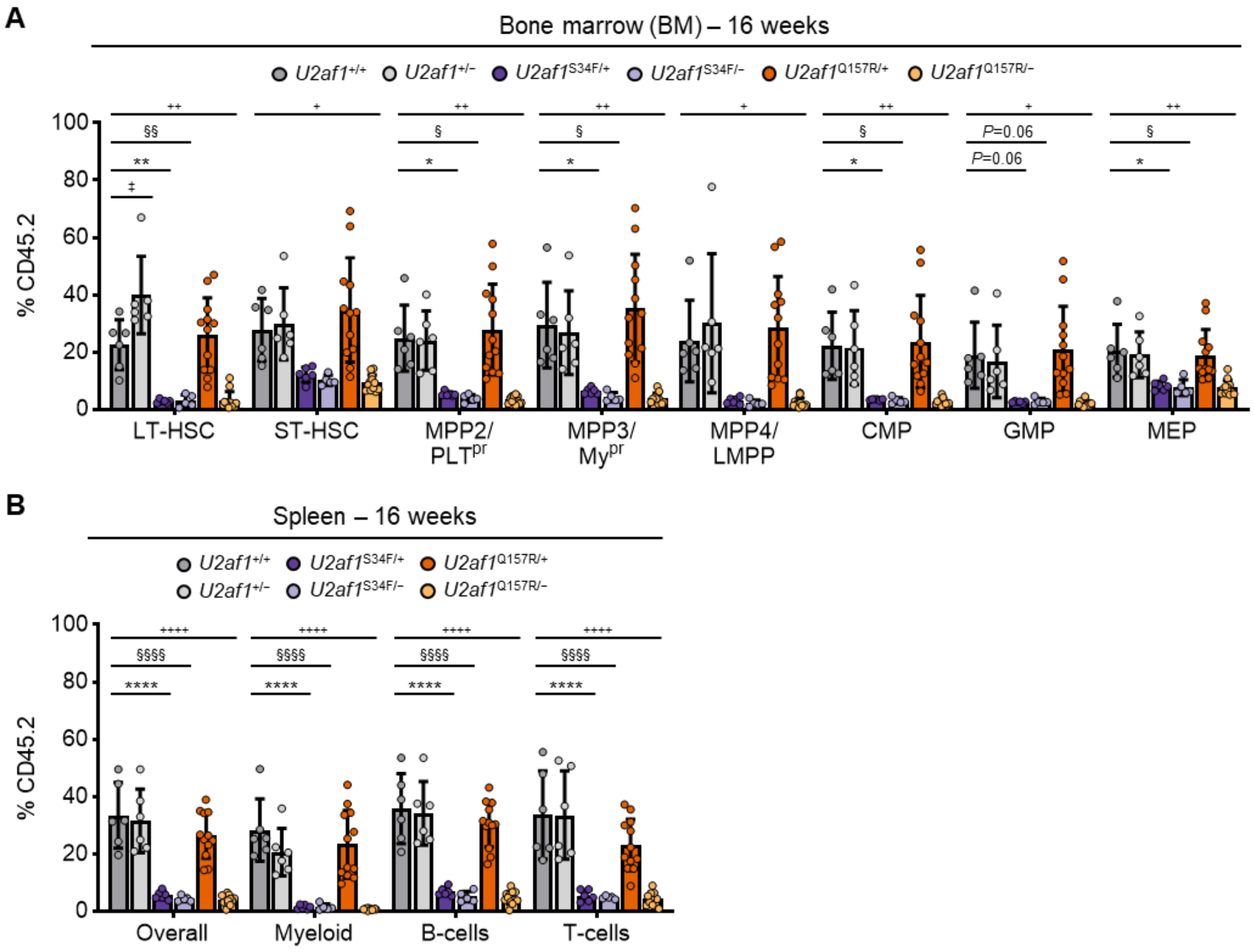

Supplementary Fig. 4. Related to Fig. 4. (**A**) Donor cell chimerism ($CD45.2^+$) of BM HSPC populations from recipient mice at 16 weeks post-pIpC. (**B**) Donor cell chimerism ($CD45.2^+$) of overall, myeloid ($CD11b^+$ cells), and lymphoid (B-cells or T-cells) splenocyte subsets from recipient mice at 16 weeks post-pIpC. Analysis was performed on the same N=6-12 randomized mice from each genotype group, as described in **Fig. 4**. Data from one *U2af1*$^{S34F/–}$ mouse was identified as a significant outlier (Grubb's test, *P* < 0.05) and removed from final analysis. Results represent the mean ± SD (**A**-**B**). A one-way ANOVA with Tukey multiple comparison correction was used for the comparison of groups (**A**-**B**). **P* < 0.05; ***P* < 0.01; *****P* < 0.0001. ns, not significant (or labeled if *P* < 0.10). Symbols (*U2af1*$^{+/+}$ vs *U2af1*$^{S34F/+}$ [*]; *U2af1*$^{+/+}$ vs *U2af1*$^{Q157R/+}$ [#]; *U2af1*$^{+/+}$ vs *U2af1*$^{S34F/–}$ [§]; *U2af1*$^{+/+}$ vs *U2af1*$^{Q157R/–}$ [+]; *U2af1*$^{+/+}$ vs *U2af1*$^{+/–}$ [‡]) are used to differentiate comparisons.

Supplementary Fig. 5

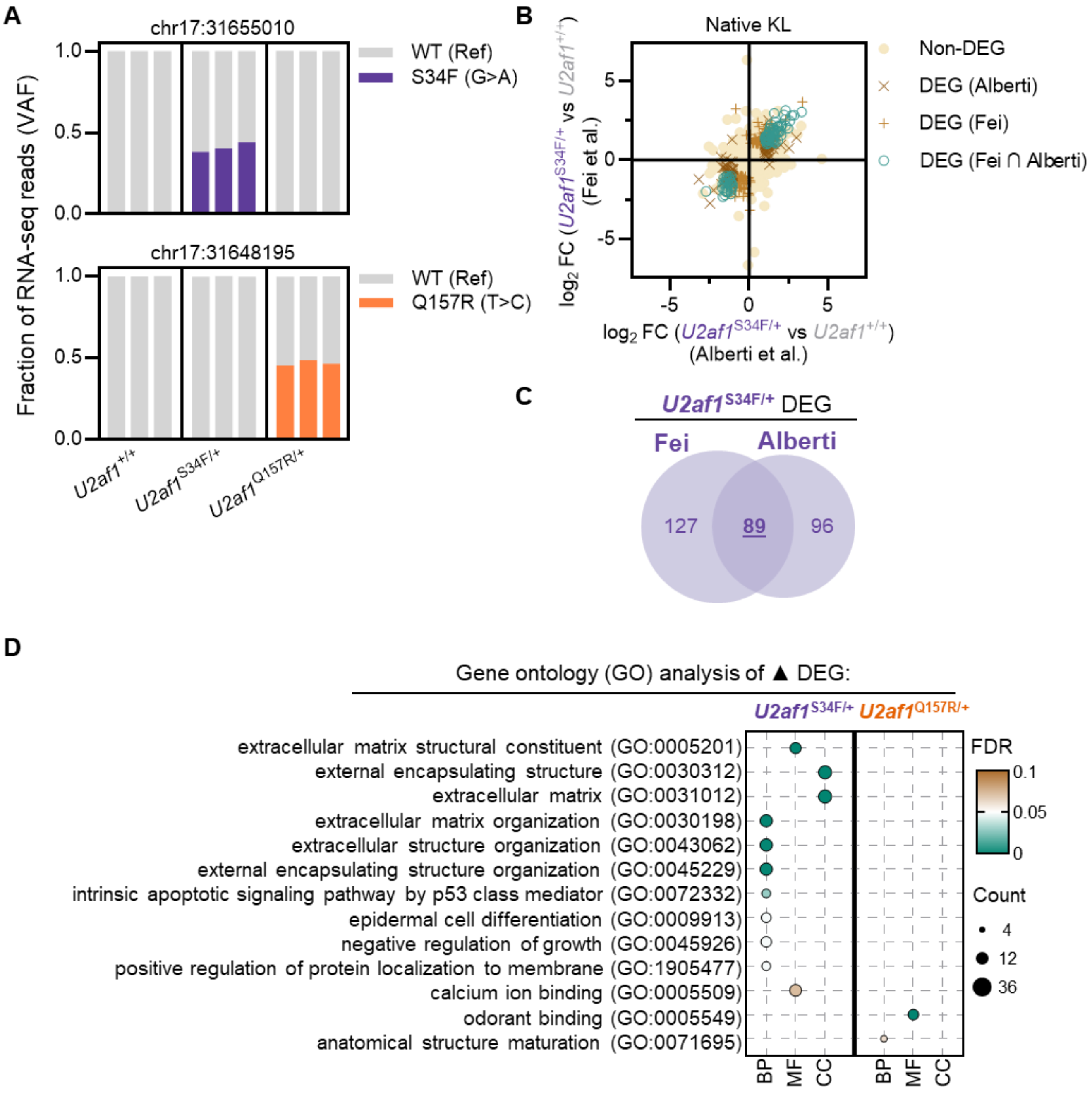

Supplementary Fig. 5

E

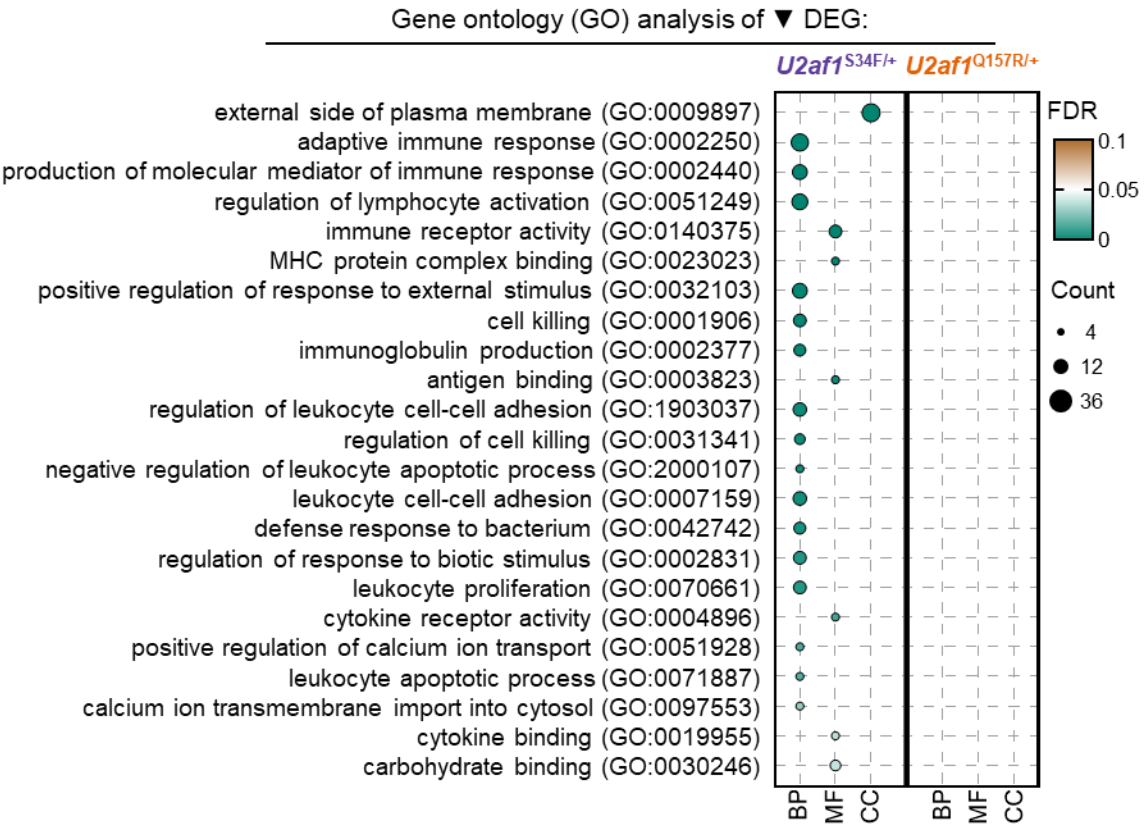

Supplementary Fig. 5. Related to Figure 5. (**A**) Variant allele frequencies (VAF) of S34F and Q157R mutations in *U2af1* mRNA from BM KL cell RNA-seq data (N=3 mice per genotype). Ref, reference. (**B**) Intersection of $\log_2$ FC values (*U2af1*$^{S34F/+}$ vs *U2af1*$^{+/+}$) for all expressed genes in Native KL (this study) and Native KL (from Fei *et al.*[1]) datasets for *U2af1*$^{S34F/+}$. Fei *et al.*[1] RNA-seq data (GSE112174) were reanalyzed for this study (N=4 KL cell samples per genotype). Genes were classified as either differentially expressed genes (DEG; FDR<0.05 and $|\log_2 \text{FC}|>1$) in one or both KL datasets or non-DEG (FDR≥0.05 and/or $|\log_2 \text{FC}|\leq 1$) in both datasets. All shared DEG were concordantly dysregulated (i.e., unidirectional $\log_2$ FC values). (**C**) Intersection of *U2af1*$^{S34F/+}$ DEG from Fei *et al.*[1] and this study (Alberti). (**D**-**E**) Gene ontology (GO) analysis of *U2af1*$^{S34F/+}$ (left) and *U2af1*$^{Q157R/+}$ (right) KL cell upregulated (**D**) and downregulated (**E**) DEGs relative to *U2af1*$^{+/+}$ KL cells. Circle size is proportional to the gene count for each term and the color indicates the magnitude of the FDR (q-value). REVIGO was used to consolidate 19 (S34F) or 12 (Q157R) gene sets from upregulated DEGs and 185 (S34F) gene sets from downregulated DEGs into representative subsets of GO terms with gene counts ≥4.[11] All significant GO terms (including for downregulated DEGs) are listed in **Supplementary Table 4**.

Supplementary Fig. 6

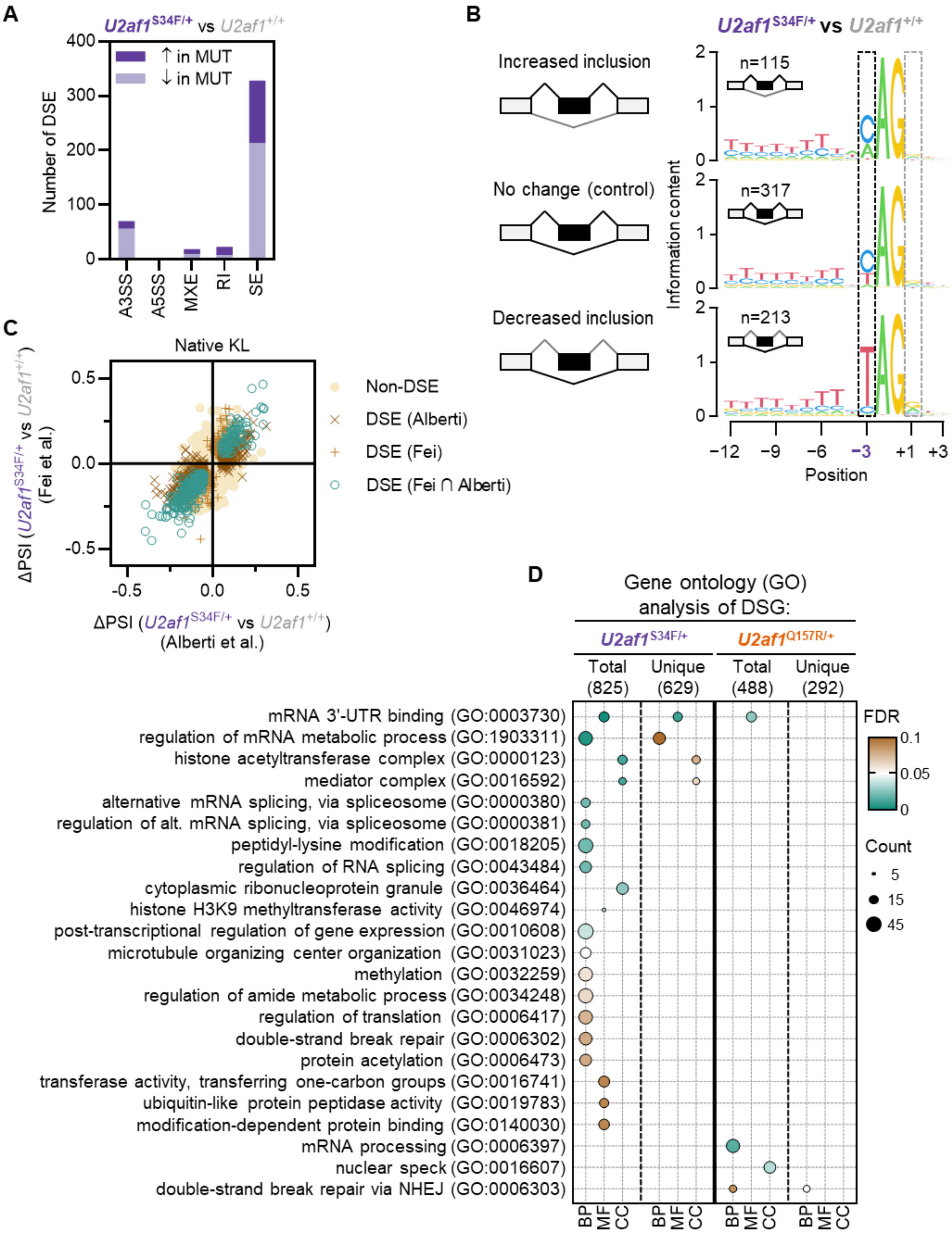

Supplementary Fig. 6. Related to Fig. 6. (**A-B**) Assessment of differential alternative pre-mRNA splicing in BM KL cells from *U2af1*$^{+/+}$ and *U2af1*$^{S34F/+}$ mice under Native conditions in Fei *et al*.[1] RNA-seq data (GSE112174) were reanalyzed for this study. N=4 KL cell samples per genotype. (**A**) Number and type (alternative 3' or 5' splice sites [A3'SS, A5'SS], mutually exclusive exons [MXE], retained introns [RI], and skipped exons [SE]) of differentially spliced events (DSE; FDR<0.05 and |ΔPSI|>0.05 vs *U2af1*$^{+/+}$) in KL cells from *U2af1*$^{S34F/+}$ mice. (**B**) Analysis of consensus 3' splice site (3'SS) sequences from control (i.e., no change in mutant vs *U2af1*$^{+/+}$) and differentially spliced SE events in *U2af1*$^{S34F/+}$ KL cells. (**C**) Intersection of ΔPSI values (*U2af1*$^{S34F/+}$ vs *U2af1*$^{+/+}$) for all annotated alternative splicing events in KL cells from Fei *et al*.[1] and this study (Alberti). Events were classified as either differentially spliced events (DSE; FDR<0.05 and |ΔPSI|>0.05) in one or both KL datasets or non-DSE (FDR≥0.05 and/or |ΔPSI|≤0.05) in both datasets. All shared DSE were concordantly dysregulated (i.e., unidirectional ΔPSI values). (**D**) Gene ontology (GO) analysis of *U2af1*$^{S34F/+}$ (left) and *U2af1*$^{Q157R/+}$ (right) KL cell DSGs relative to *U2af1*$^{+/+}$ KL cells. GO was performed on the total number of DSGs identified in each mutant as well as on the DSGs specific (unique) to each mutant. Circle size is proportional to the gene count for each term and the color indicates the magnitude of the FDR (q-value). REVIGO was used to consolidate 61 (S34F) or 6 (Q157R) gene sets from total DSGs and 7 (S34F) and 1 (Q157R) gene sets from unique DSGs into representative subsets of GO terms with gene counts ≥5.[11] All significant GO terms are listed in **Supplementary Table 9**.

Supplementary Fig. 7

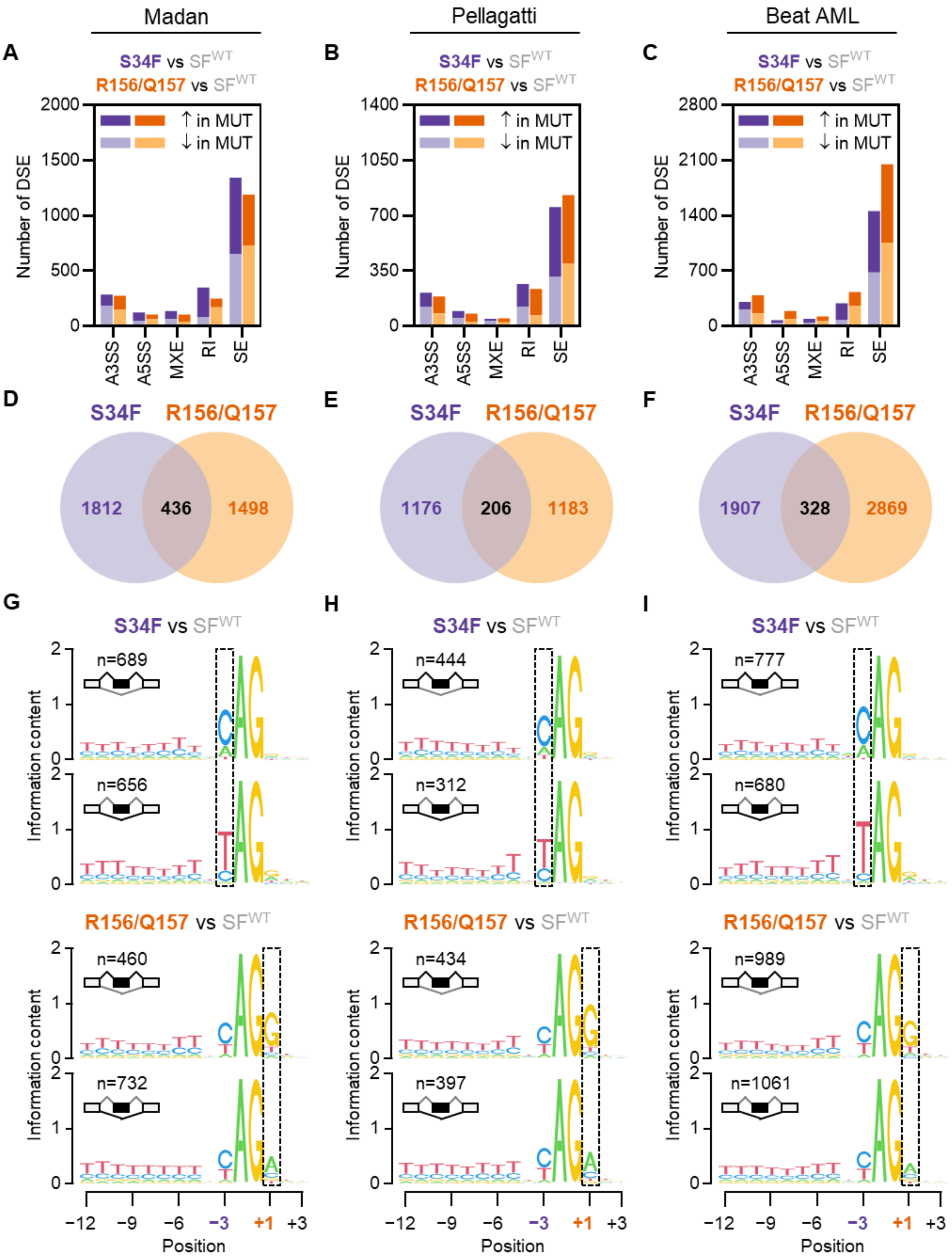

Supplementary Fig. 7

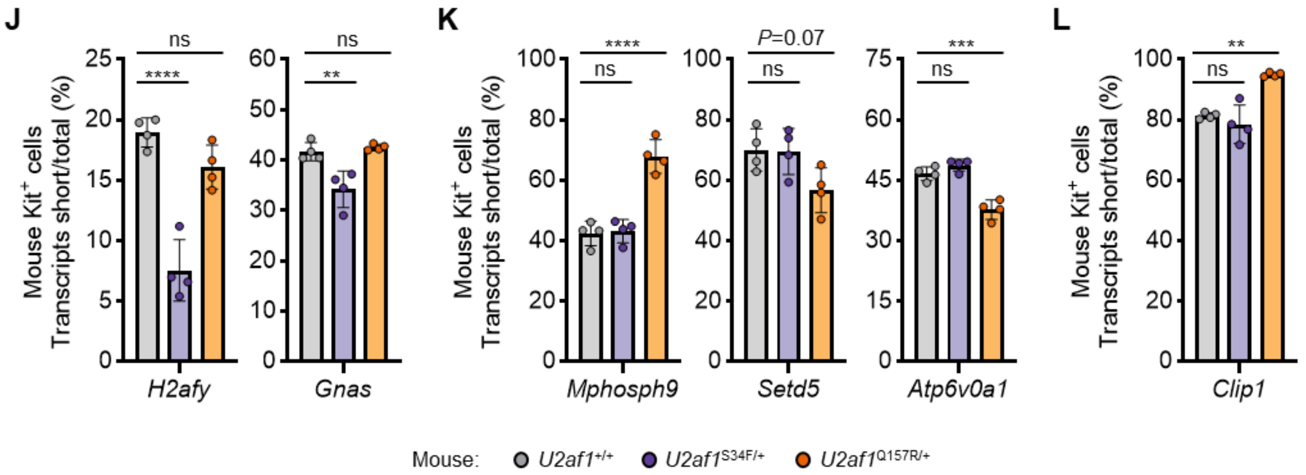

Supplementary Fig. 7. Related to Fig. 7. Analsyis of publicly available BM cell RNA-seq data from MDS (Madan [**A**, **D**, **G**] and Pellagatti [**B**, **E**, **H**]) and AML (Beat AML [**C**, **F**, **I**]) patients. RNA-seq data from Madan *et al.*[14] (GSE128429), Pellagatti *et al.*[15] (GSE114922), and Beat AML[16] (phs001657.v1.p1) were reanalyzed for this study. N=2-10 samples per mutant genotype per study. N=8 (Madan), 40 (Pellagatti), or 279 (Beat AML) SF^{WT} samples. (**A**-**C**) Number and type (alternative 3' or 5' splice sites [A3'SS, A5'SS], mutually exclusive exons [MXE], retained introns [RI], and skipped exons [SE]) of differentially spliced events (DSE; FDR<0.05 and |ΔPSI|>0.05 vs splicing factor WT [SF^{WT}] patients) in BM cells from MDS and AML patients harboring *U2AF1*^{S34F} (S34F) or *U2AF1*$^{R156H/Q157(P/R)}$ (R156/Q157) mutations. (**D**-**F**) Overlap of DSE in BM cells from MDS and AML patients harboring S34F or R156/Q157 mutations. (**G**-**I**) Analysis of consensus 3' splice site (3'SS) sequences from differentially spliced SE events in S34F or R156/Q157 BM cells from MDS and AML patients. The highlighted −3 and +1 positions of the 3'SS recapitulate the aberrant consensus 3'SS sequence dependencies identified previously in *U2AF1*^{S34F} and *U2AF1*^{Q157} MDS patients. (**J**-**L**) RT-PCR orthogonal confirmation of S34F or Q157R aberrantly spliced transcripts in mutant mouse Kit^{+} BM cells (24 weeks post-pIpC). Quantification of aberrantly spliced transcripts in S34F (**J**), Q157P/R (**K**), or both (**L**). Results represent the mean ± SD (**J**-**L**). A one-way ANOVA with Tukey multiple comparison correction (**J**-**L**) was used for the comparison of groups. $^{**}P < 0.01$; $^{***}P < 0.001$; $^{****}P < 0.0001$. ns, not significant (or labeled if $P < 0.10$).

Supplementary Fig. 8

A

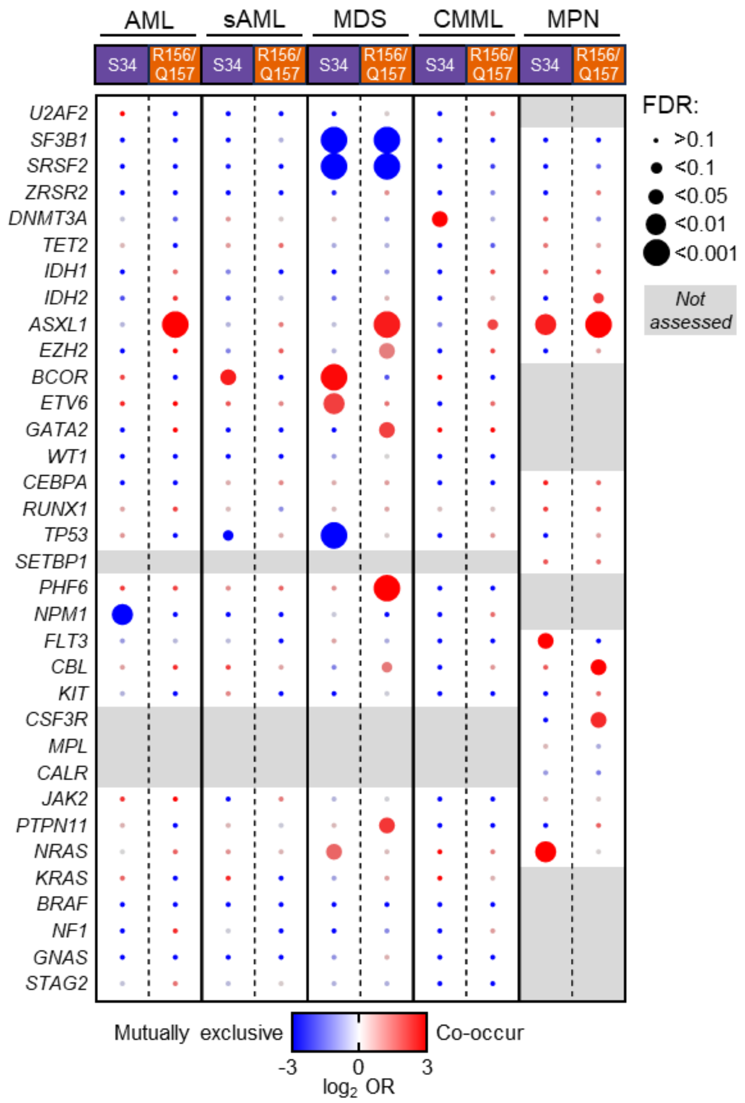

Supplementary Fig. 8

B

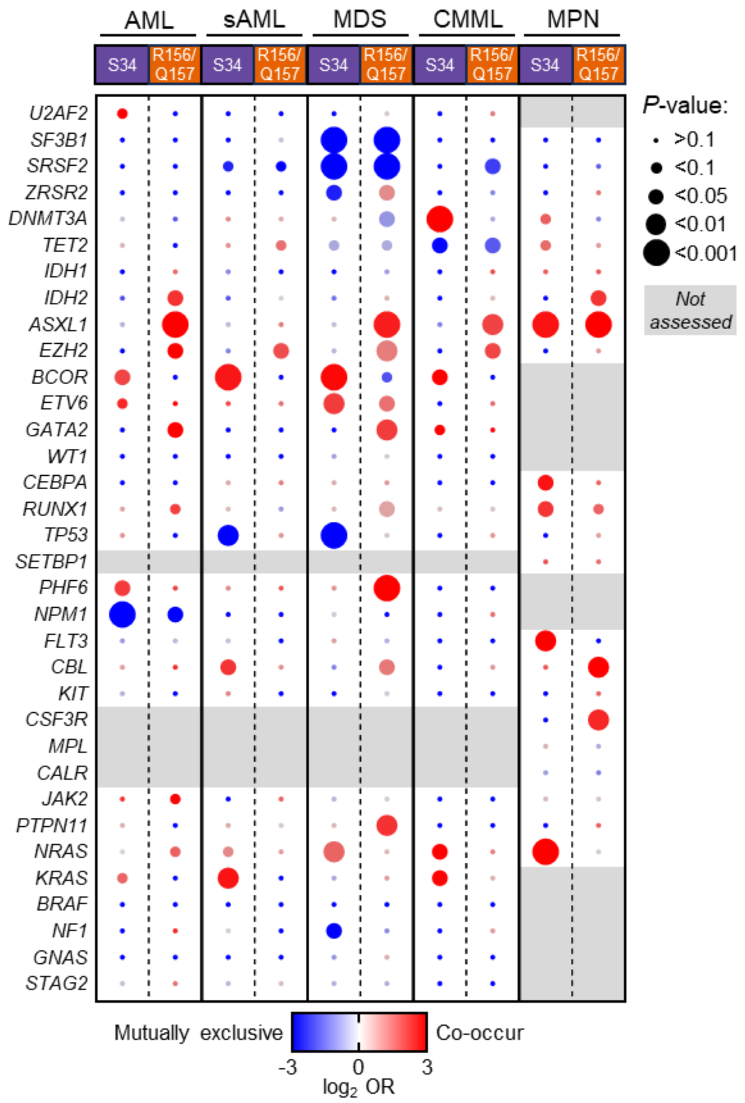

Supplementary Fig. 8

c

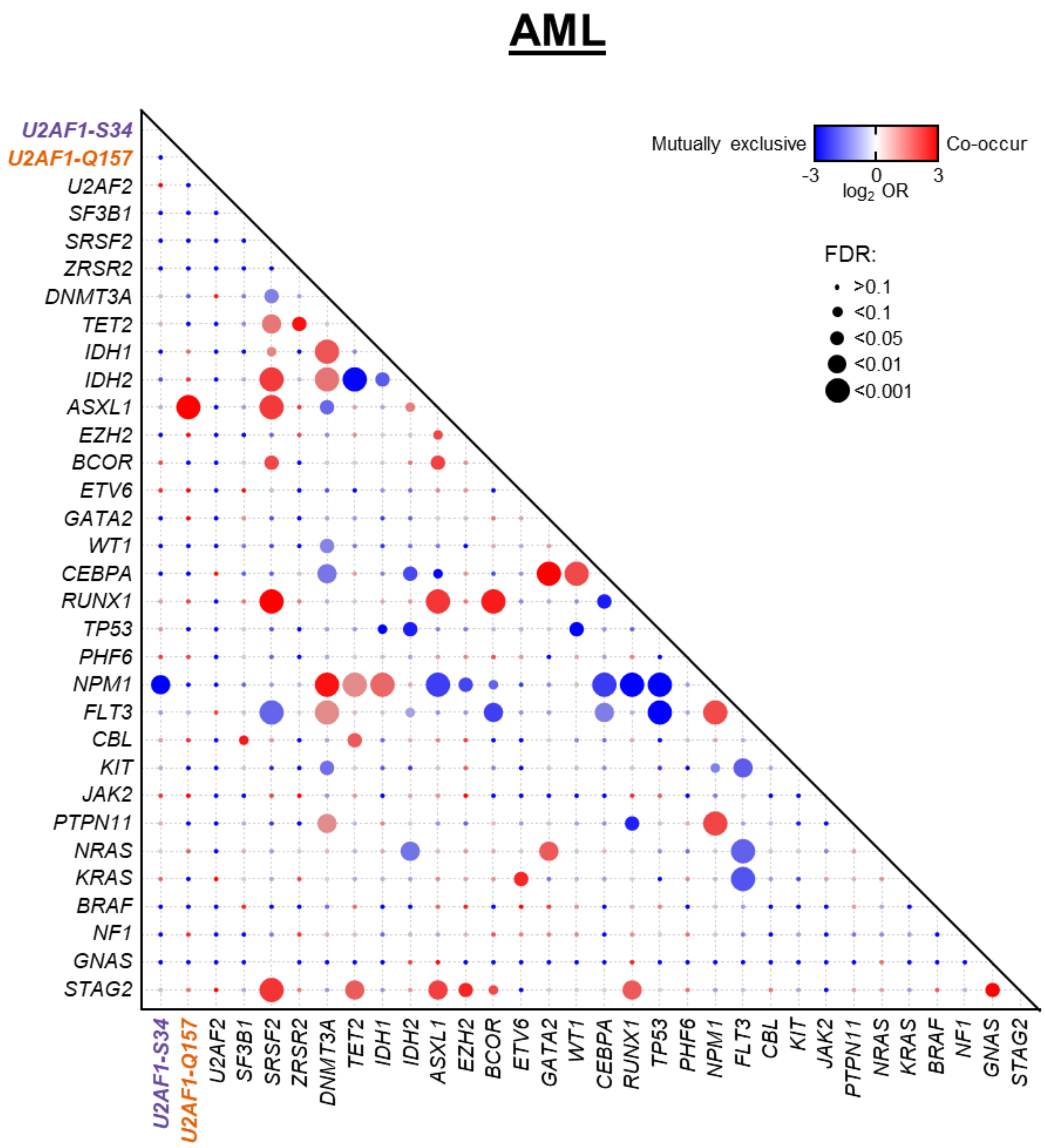

Supplementary Fig. 8

D

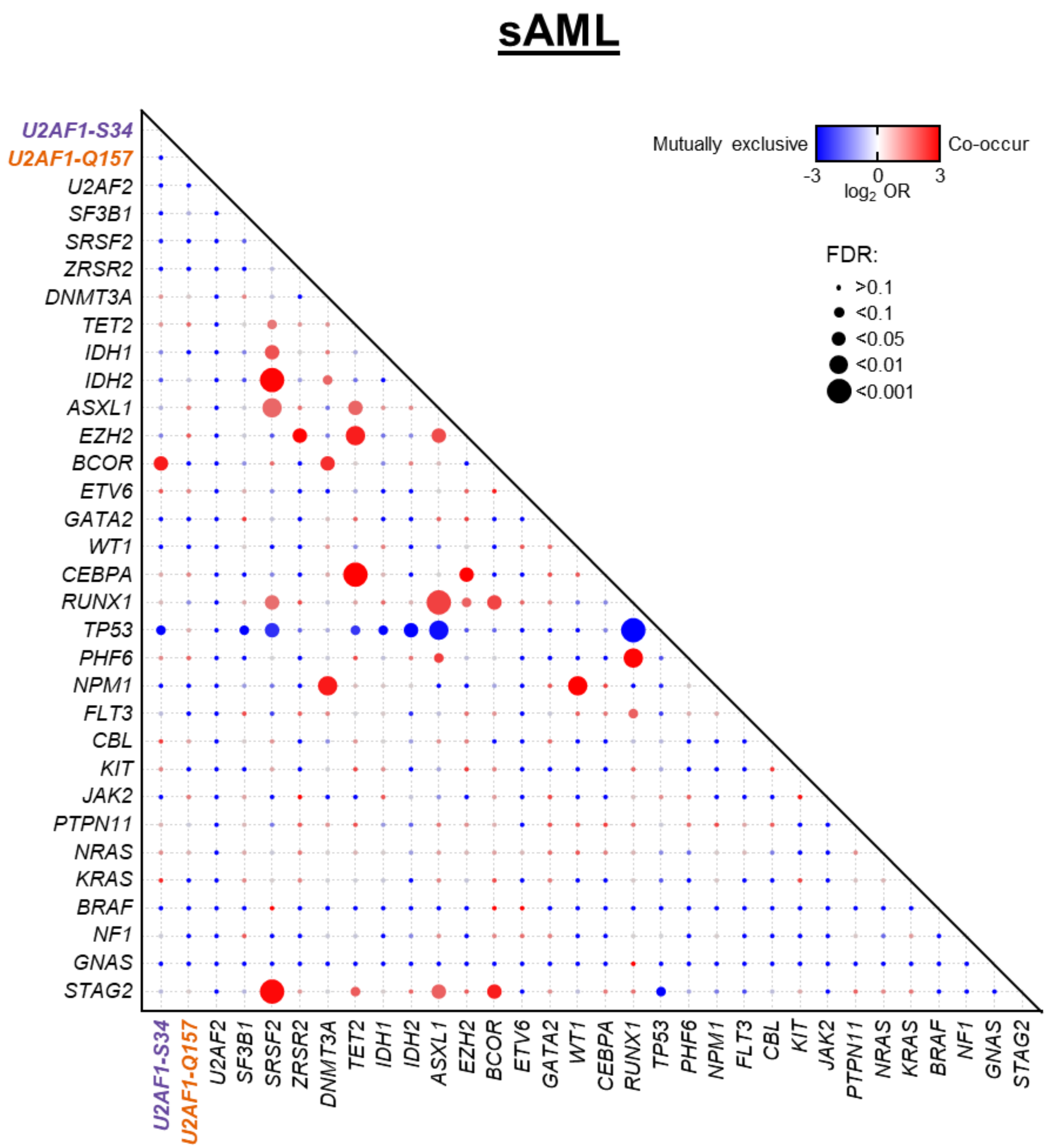

Supplementary Fig. 8

E

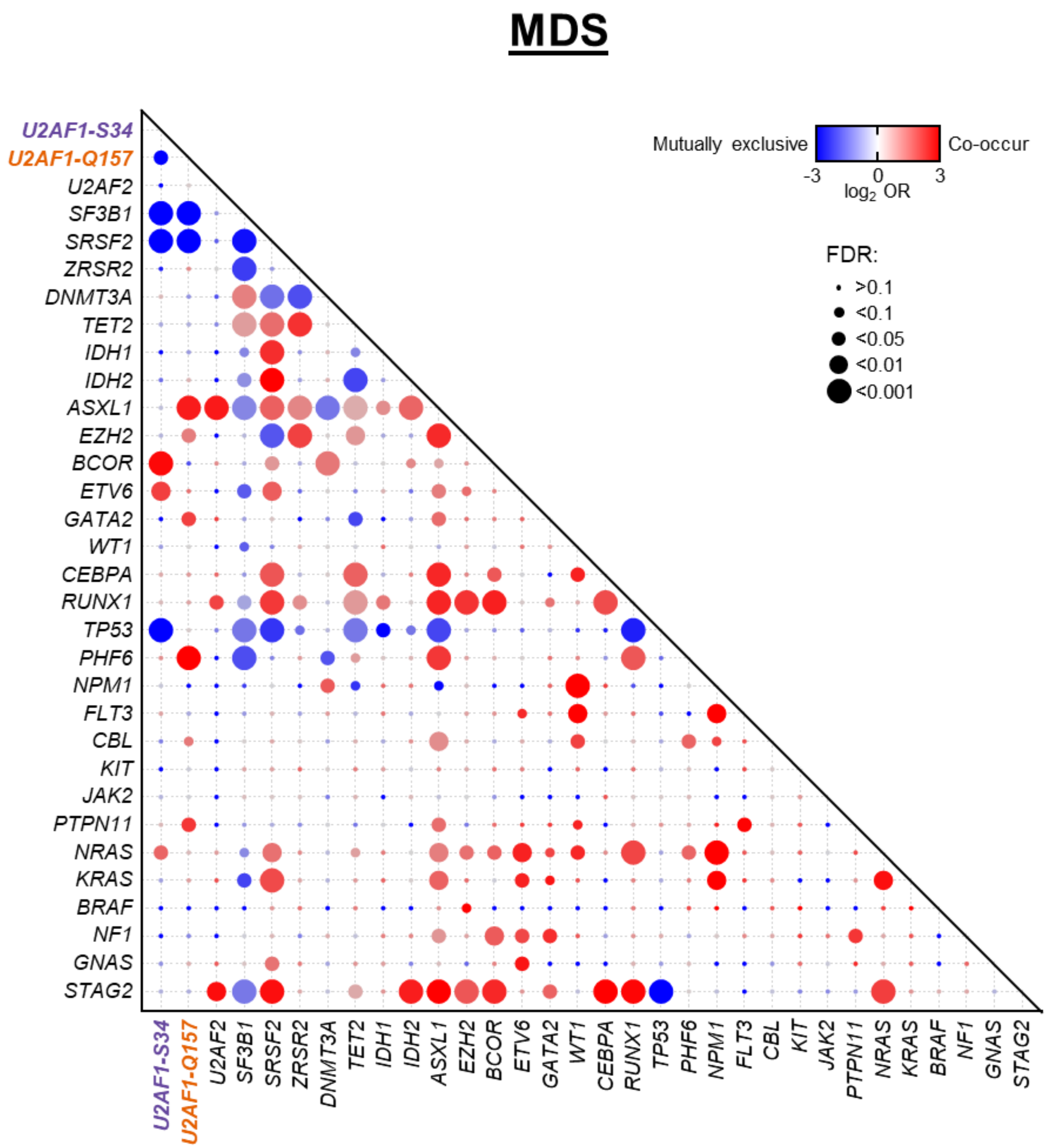

Supplementary Fig. 8

F

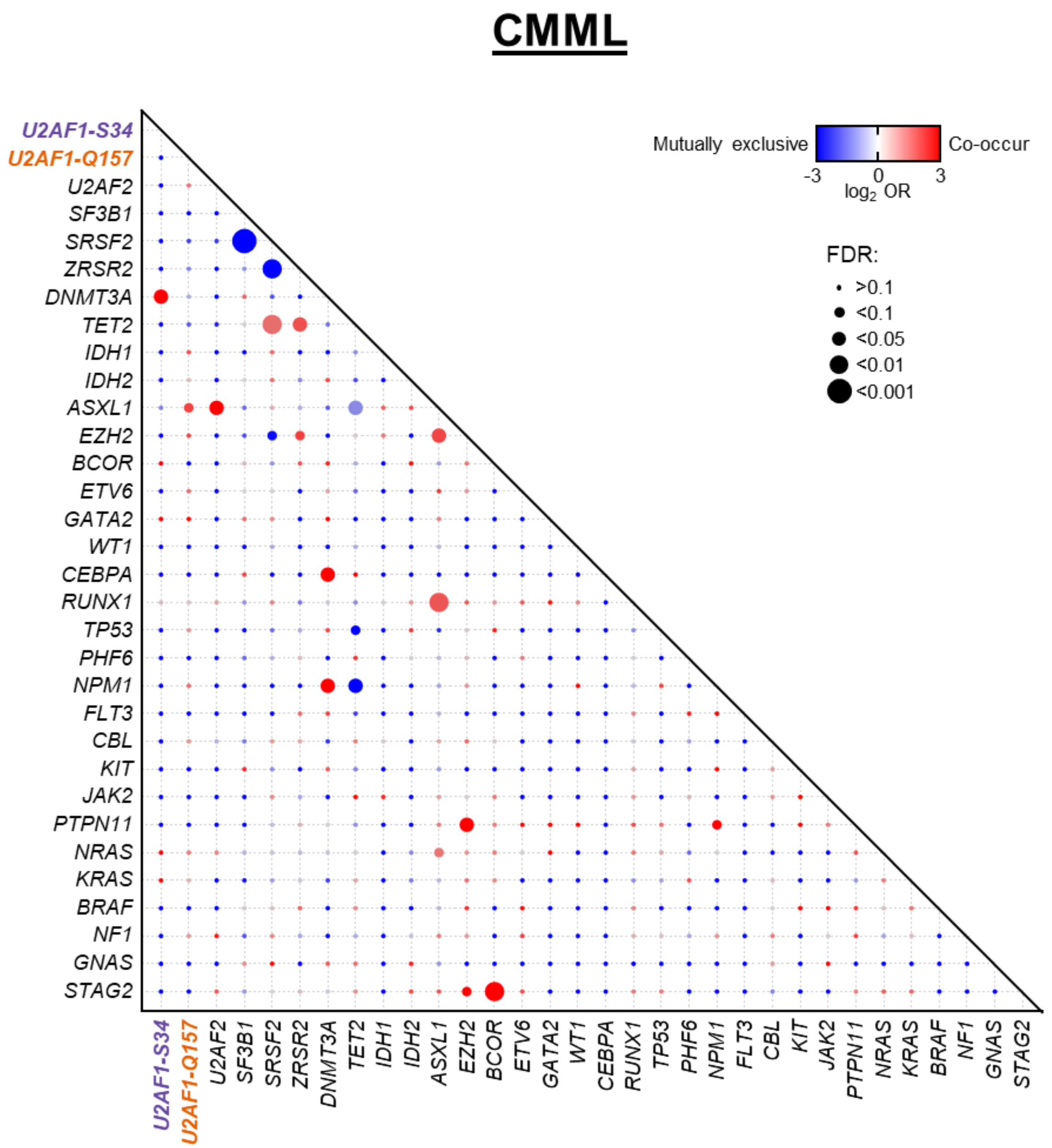

Supplementary Fig. 8

G

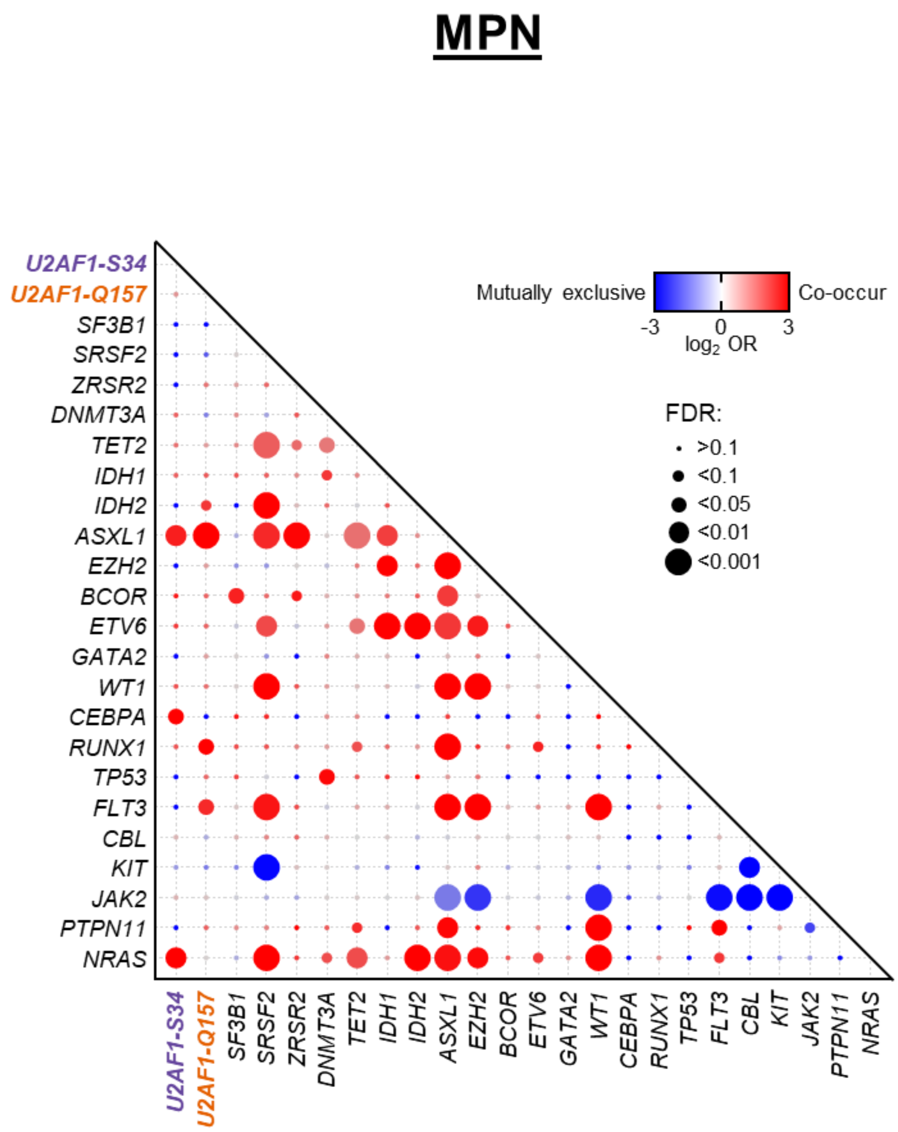

Supplementary Fig. 8. Related to Fig. 8. (**A-G**) Analysis of *U2AF1* hotspot mutation co-occurrence and mutual exclusivity in myeloid malignancies. Mutation data for patients with a diagnosis of AML (N=1857 patients; **A-C**), sAML (from MDS; N=458 patients; **A**,**B**,**D**), MDS (N=3159 patients; **A**,**B**,**E**), CMML (N=430 patients; **A**,**B**,**F**), and MPN (N=1551 patients; **A**,**B**,**G**) were included from 20 published studies that performed *U2AF1* sequencing and had patient-level mutation data available for a common set of 23 (MPN) or 31 (AML, sAML, MDS, and CMML) genes sequenced across all studies (see **Supplementary Methods** and **Supplementary Tables 19-20**). cBioPortal was used for the co-occurrence and mutual exclusivity of genomic alteration analysis within each disease group using the default settings. Circle color indicates the $\log_2$ odds ratio (OR) for each gene pair and size is proportional to the magnitude of either the FDR (q-value; **A**,**C-G**) or *P*-value (**B**).
